# Structural Basis for Cyclosporin Isoform-Specific Inhibition of Cyclophilins from *Toxoplasma gondii*

**DOI:** 10.1021/acsinfecdis.2c00566

**Published:** 2023-01-18

**Authors:** Filippo Favretto, Eva Jiménez-Faraco, Carolina Conter, Paola Dominici, Juan A. Hermoso, Alessandra Astegno

**Affiliations:** † Department of Biotechnology, University of Verona, Strada Le Grazie 15, 37134Verona, Italy; ‡ Department of Crystallography and Structural Biology, Institute of Physical Chemistry Rocasolano (IQFR), CSIC, Serrano 119, 28006Madrid, Spain

**Keywords:** Toxoplasma gondii, cyclophilin, cyclosporin, peptidyl-prolyl isomerization, chaperone-like activity, crystal structure

## Abstract

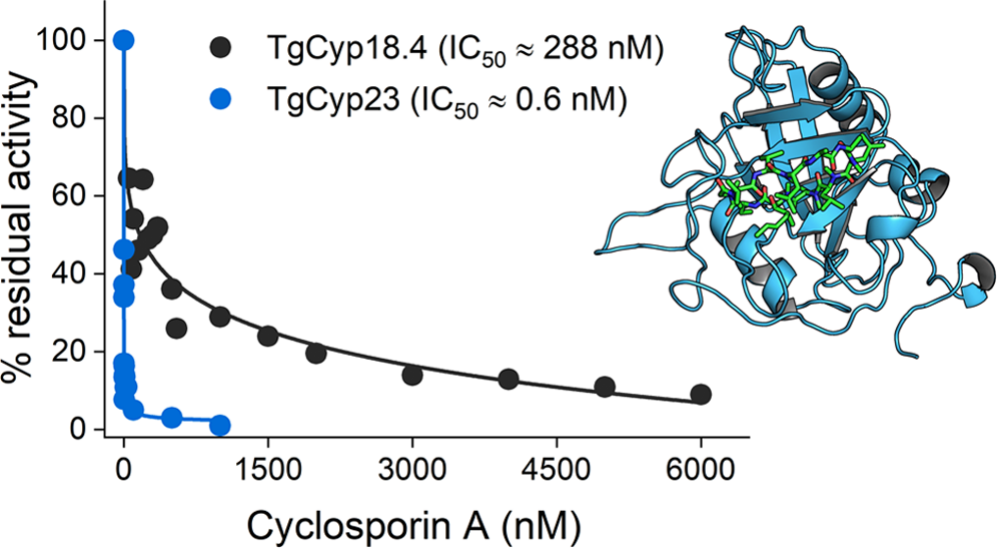

Cyclosporin (CsA) has antiparasite activity against the human pathogen *Toxoplasma gondii*. A possible mechanism of action involves CsA binding to *T. gondii* cyclophilins, although much remains to be understood. Herein, we characterize the functional and structural properties of a conserved (TgCyp23) and a more divergent (TgCyp18.4) cyclophilin isoform from *T. gondii*. While TgCyp23 is a highly active cis–trans-prolyl isomerase (PPIase) and binds CsA with nanomolar affinity, TgCyp18.4 shows low PPIase activity and is significantly less sensitive to CsA inhibition. The crystal structure of the TgCyp23:CsA complex was solved at the atomic resolution showing the molecular details of CsA recognition by the protein. Computational and structural studies revealed relevant differences at the CsA-binding site between TgCyp18.4 and TgCyp23, suggesting that the two cyclophilins might have distinct functions in the parasite. These studies highlight the extensive diversification of TgCyps and pave the way for antiparasite interventions based on selective targeting of cyclophilins.

Cyclophilins (Cyps) are ubiquitous and evolutionarily well-conserved proteins first recognized as specific cellular receptors for the immunosuppressant cyclosporin A (CsA). These proteins are endowed with peptidyl-prolyl isomerase (PPIAse) activity to catalyze trans-to-cis isomerization of peptide bonds at proline residues, which is a critical step for correct protein folding.^1^ Cyps appear to be involved in several key processes, including signal transduction, cell differentiation, RNA processing, protein secretion, and protein trafficking.^2−5^ Along with PPIase activity, some Cyps also show an independent chaperone activity, supporting their versatile properties.^6−9^

Genome-wide analysis has identified a diverse number of Cyp genes in different organisms, ranging from 17 in the human genome,^10^ 11 in *Caenorhabditis elegans*,^11^ at least nine in *Drosophila melanogaster*,^12^ and eight in *Saccharomyces cerevisiae.*(13,14) Interestingly, larger Cyp gene families have been found in plants (e.g., 29 members in *Arabidopsis thaliana*, 27 in rice, 62 in soybean), but the functions of most plant Cyps are still elusive.^15−18^ All Cyps possess a so-called cyclophilin-like domain (CLD) with a typical architecture comprising eight antiparallel β-strands sandwiched between two helices. Moreover, some Cyps are extended by additional structural elements and domains (e.g., WD domain, Leu zipper, phosphatase binding domain, TPR domain), and for these multidomain Cyps, distinct roles in protein–protein interactions have been hypothesized.^19^

The prototypical member of the Cyp family is the human CypA, a single-domain 18 kDa protein that mainly mediates the action of the immunosuppressive drug CsA. While CsA can inhibit the PPIase activity of Cyp with high potency, the main immunosuppressive effect arises from the formation of a complex with Cyp. This complex has a nanomolar affinity and inhibits the calcium-dependent phosphatase calcineurin, therefore preventing its translocation to the nucleus and activation of T-cells.^20,21^ The reduction of T-cell activity by CsA has made it extremely useful in organ transplantation and management of various autoimmune diseases.^2^ For their involvement in immune response, some Cyps are also named immunophilins.

In addition to an immunosuppressive effect, CsA has other activities including antiparasitic activity against various pathogens (reviewed in refs (22−24)). For example, in malarial mouse models, CsA significantly inhibits the growth of the parasite.^25^ Moreover, CsA has a leishmanicidal effect on intracellular *Leishmania tropica* and *Leishmania major* models.^26−28^ CsA activity against *Toxoplasma gondii* parasites, the causative agent of toxoplasmosis, has also been described.^24^ However, even if the impact of CsA on parasites of public health importance has been highlighted, current research on the mechanisms of the antiparasitic CsA still warrants further study. In analogy with the human system, CsA-binding parasite Cyps are potential drug targets. In this scenario, the first step in understanding the antiparasitic activity of CsA is by gaining knowledge of the potential receptor molecules for CsA.

*T. gondii* harbors 15 genes that encode putative Cyps,^29^ meaning Cyps in the parasite may have various biological functions. However, to date, only one *Toxoplasma* Cyp has been characterized, i.e., TgCyp18, which appears to have a crucial role in the modulation of pro- and anti-inflammatory signals that regulate parasite migration in the host.^30,31^

In this work, we investigated the molecular properties of two recombinant Cyps from *T. gondii* to elucidate their PPIase and chaperone activities, their ability to bind CsA, and their sensitivity to the drug. We found that both Cyps are functional in PPIase assays but significantly differ in their catalytic efficiency and CsA inhibition. Moreover, we determined the crystal structure of TgCyp23 in complex with CsA at the atomic resolution showing the molecular details of CsA recognition by the protein and explaining the experimental CsA binding parameters. Structural comparison between CsA recognition in TgCyp18.4 and TgCyp23 revealed relevant differences in the CsA-binding pocket and the change of some critical residues that could explain the lower CsA binding values observed for TgCyp18.4.

These results have relevant implications for elucidating the mechanism of the anti-*Toxoplasma* action of CsA, supporting anti-*Toxoplasma* interventions based on selective pharmacological targeting of Cyps.

## Results

**Figure 1:**
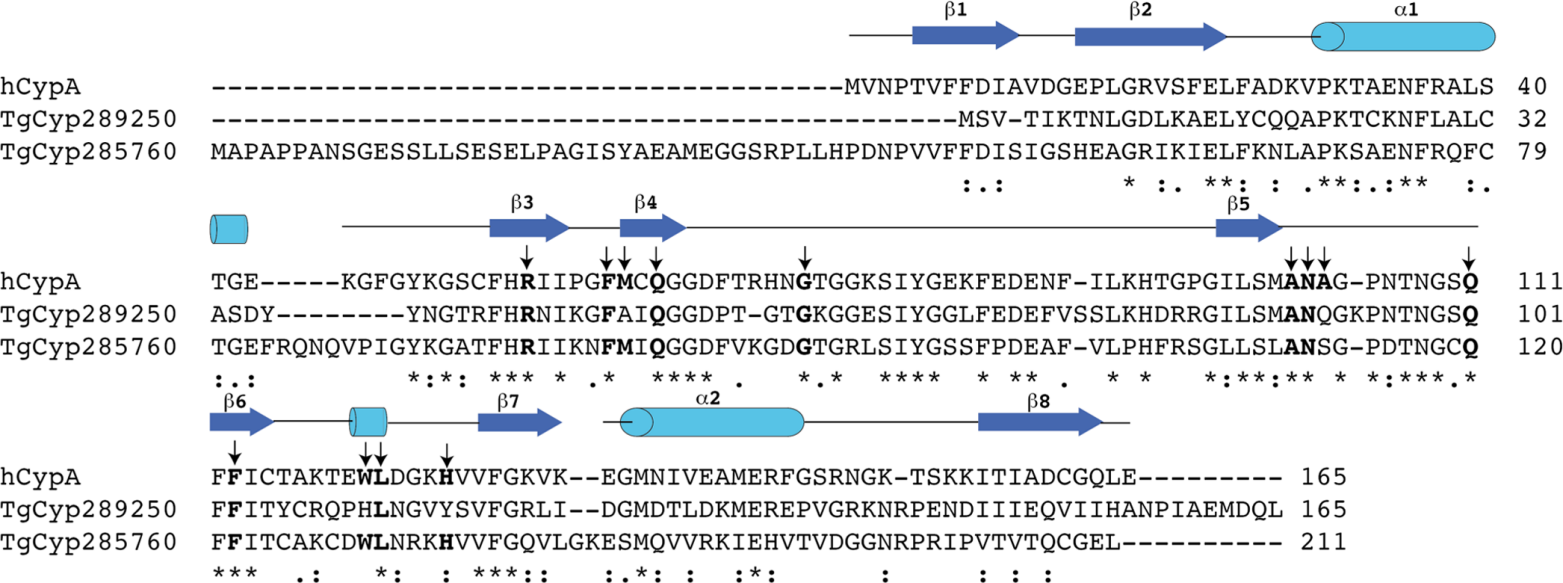
Sequence alignment of TgCyp18.4, TgCyp23, and human CypA. Amino acids critical for Cyp enzymatic activity and CsA binding are shown in bold. The sequences shown are TgCyp18.4 (TgCyp289250), TgCyp23 (TgCyp285760), and human CypA.

To identify Cyps from *T. gondii*, we searched the NCBI database for homologues of human CypA. Thirteen Cyp homologues were identified, and their amino acid sequences were aligned using Clustal Omega (Figure S1). The length of the protein homologues identified ranged from 165 to 764 amino acids, with many members of the family containing large N- and C-terminal extensions relative to human CypA, thus suggesting the presence of larger multidomain proteins containing one or several unrelated domains in addition to the typical cyclophilin-like domain (CLD). The primary sequences of the identified proteins share 28–69% identity to human CypA. Of note, the CLD of most *Toxoplasma* Cyp members contains several active site residues that are highly conserved in the cyclophilin family (e.g., Arg55, Phe60, Gln63, Gly72, Ala101, Asn102, Phe113, Leu122, His126, following the nomenclature of human CypA). In this work, we have selected TgCyp285760 (named TgCyp23) and TgCyp289250 (named TgCyp18.4) for further biochemical and structural characterization as representatives of conserved as well as more divergent TgCyps candidates since they have varying degrees of active site residue identity to the well-studied human CypA. In particular, the sequence comparison of TgCyp23 with human CypA suggested that, among the 13 residues characterizing the CsA-binding pocket in the human CypA–CsA complex (Arg55, Phe60, Met61, Gln63, Gly72, Ala101, Asn102, Ala103, Gln111, Phe113, Trp121, Leu122, His126), 12 are identical in TgCyp23, including a single tryptophan that is likely to be critical for CsA binding. However, TgCyp23 possesses a 40-residue N-terminal extension relative to human CypA (Figure 1) that might be involved in the membrane interaction and subcellular localization in agreement to other parasitic systems.^32^ On the other hand, TgCyp18.4 contains some striking amino acid substitutions in the active site (e.g., Met61, Trp121, and His126 of CypA are replaced by Ala50, His111, and Tyr116, respectively, in TgCyp18.4), which could, in principle, influence the catalytic properties of the enzyme and its affinity for CsA, making it an attractive candidate for the design of new CsA derivative compounds (Figure 1).

The two selected proteins were expressed in *E. coli* cells and purified to homogeneity. They remained highly soluble, even after TEV removal of the fusion His-tag (Figure S2A). Both TgCyp18.4 and TgCyp23 showed a well-defined and symmetric peak in the size exclusion chromatography (SEC) profile at elution volumes corresponding to apparent molecular masses of 20.9 ± 0.2 and 19.7 ± 0.2 kDa, respectively, which are in excellent agreement with both being monomeric in solution (Figure S2B).

### TgCyps Are Well-Folded in Solution

**Figure 2:**
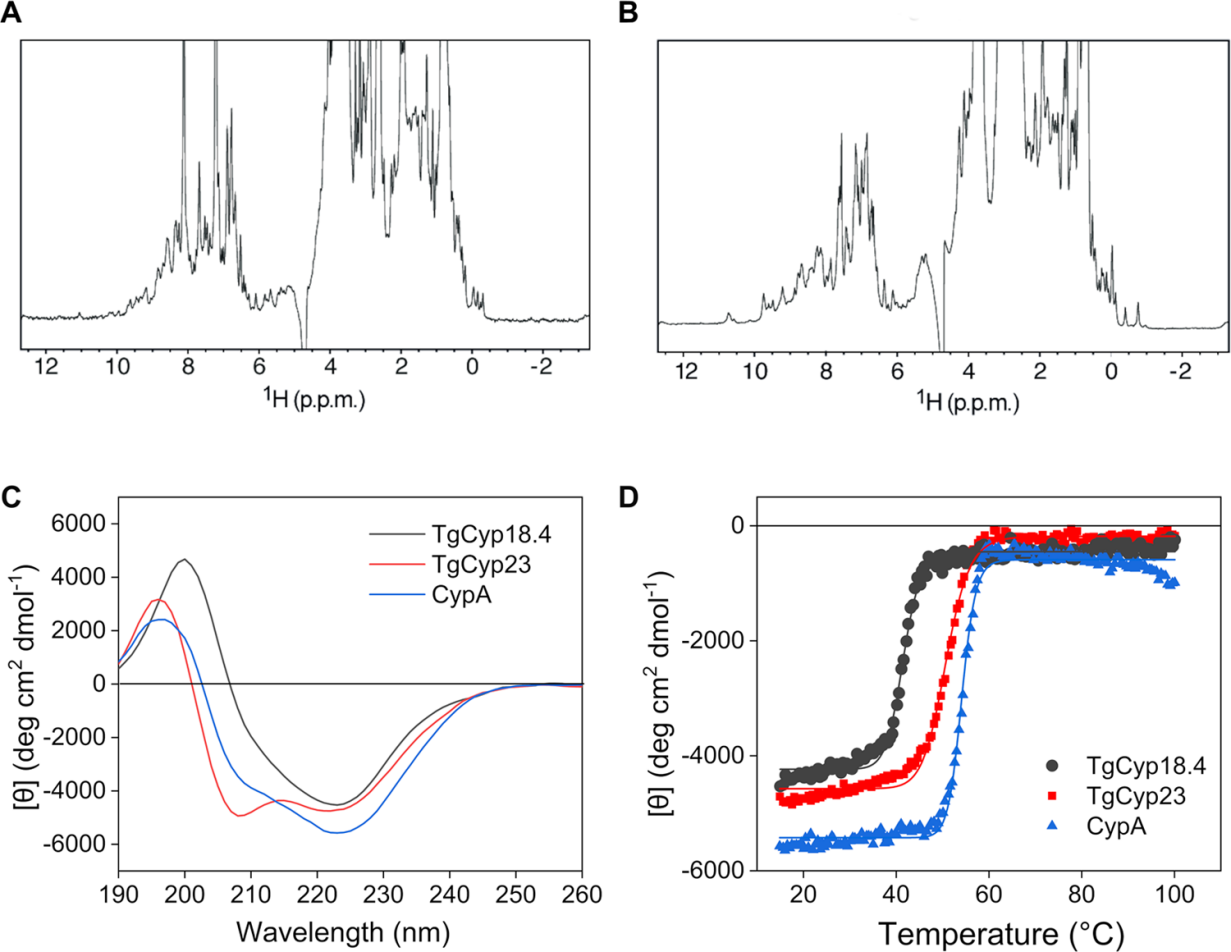
Structural characterization of TgCyp18.4 and TgCyp23. (A, B) ^1^H-1D NMR spectra of (A) 70 μM TgCyp18.4 (64 scans) and (B) 100 μM TgCyp23 (16 scans). (C) Far-UV CD spectra and (D) thermal denaturation profiles of 0.2 mg mL^–1^ TgCyp18.4 (black) and TgCyp23 (red). CypA was included as comparison (blue).

NMR and CD spectroscopy was applied to investigate the structural integrity and folding state of the two selected TgCyps. One-dimensional (1D) ^1^H NMR spectroscopy can give useful information on protein folding. A good signal dispersion of the ^1^H frequencies in the range ∼−1 to 11 ppm was detected for both Cyps, indicating that each nucleus is experiencing a well-defined chemical environment, typical of correctly folded proteins (Figure 2A,B). In addition, the presence of methyl (∼−1 to 0 ppm) and amidic proton (∼7 to 11 ppm) resonances outside the random coil chemical shift regions are also an index of correctly folded proteins, with a defined tertiary structure.

The NMR data comply with the CD experiments in the far-UV region (200–250 nm), which gives information on protein secondary structural elements (Figure 2C). The far-UV CD spectrum of TgCyp18.4 resembled that of human CypA showing a predominant content of β-sheets, followed by α-helices as expected for a Cyp protein (Table S1). On the other hand, the far-UV CD spectrum of TgCyp23 exhibited more pronounced minima at 208 and 222 nm, typical of a protein with prevalent α-helical content (Table S1). This difference could be due to the presence in TgCyp23 of the 40-residue N-terminal extension, which can have some α-helical propensity. Overall, the CD spectra show that both TgCyps are well-folded in solution.

We next assessed the thermal stability of the two proteins, monitoring the changes in the secondary structure while increasing the temperature. Thermal denaturation curves were obtained by following the CD signal at 222 nm, and the *T*_m_ was determined by fitting the measured ellipticity to a two-state transition model (Figure 2D). Notably, TgCyp18.4 was rather unstable with a *T*_m_ of 44 ± 2 °C. In contrast, for TgCyp23, a *T*_m_ of 52 ± 1 °C was found, which is more similar to that of human CypA (*T*_m_ 54 ± 1 °C) (Table 1).

**Table 1:** Thermal Stability of Cyp Variants in the Absence and Presence of CsA Assessed by Circular Dichroism (CD) and Differential Scanning Calorimetry (DSC)

protein	CD *T*_m_ (°C)	DSC *T*_m_ (°C)
CypA	54 ± 1	51 ± 1
CypA + CsA	59 ± 1	58 ± 1
TgCyp18.4	44 ± 2	40 ± 1
TgCyp18.4 + CsA	44 ± 2	39 ± 1
TgCyp23	52 ± 1	49 ± 1
TgCyp23 + CsA	55 ± 1	54 ± 1

### TgCyps Have Different PPIase Activities

To investigate the PPIase activity of the TgCyp18.4 and TgCyp23, we carried out a protease-coupled PPIase assay as described in ref (33). The method uses a tetrapeptide (*N*-succinyl-Ala-Ala-Pro-Phe-*p*-nitroanilide, AAPF) as a chromogenic substrate that has a cis conformation of the peptide bond involving a proline residue. A trans-isomer is formed by Cyp, which is selectively cleaved by chymotrypsin, producing 4-nitroaniline, a yellow-colored product that can be measured spectrophotometrically.

**Figure 3:**
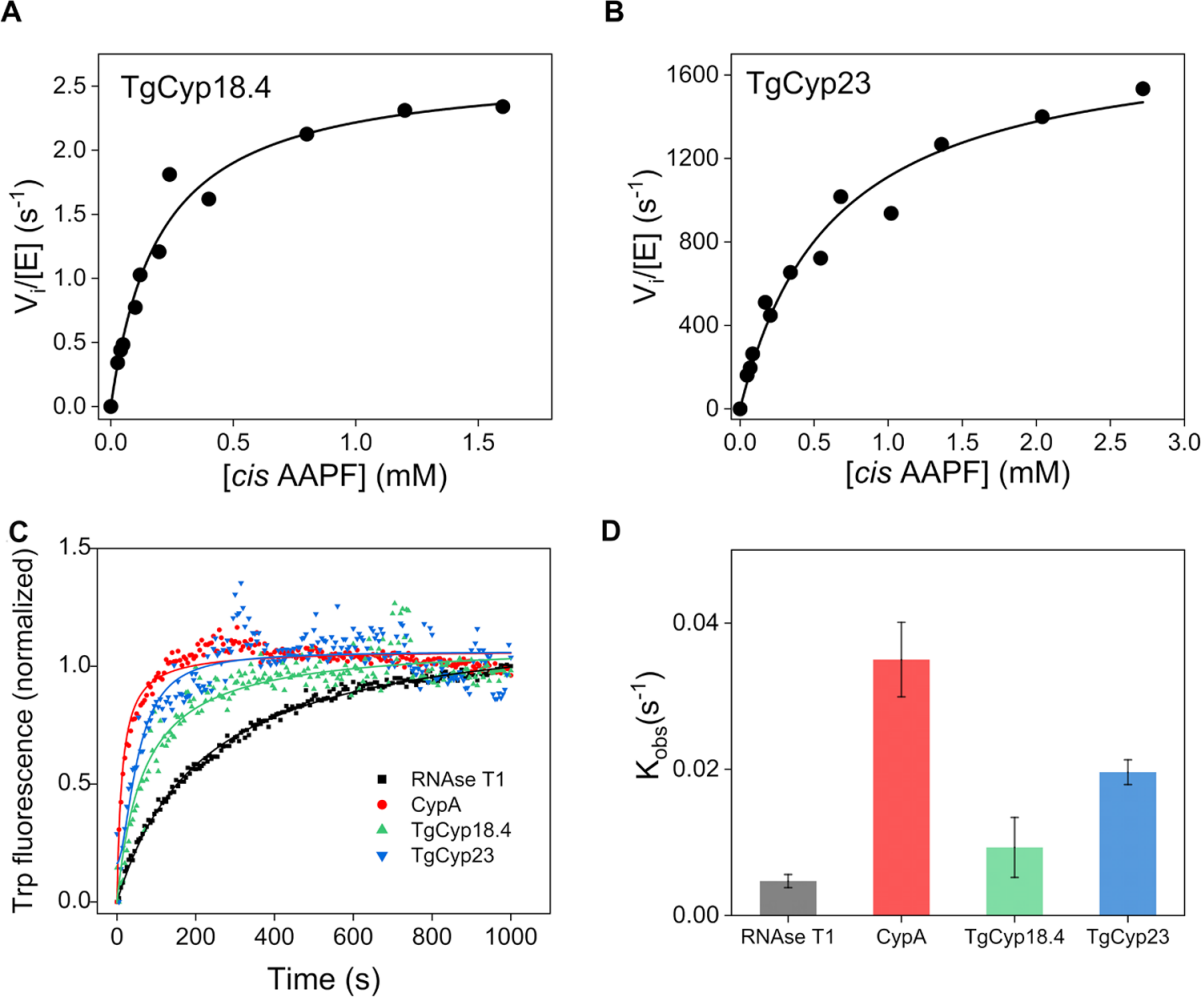
PPIase and chaperone-like activity of TgCyps. (A, B) Representative steady-state initial velocity kinetics for the PPIase activity of (A) TgCyp18.4 and (B) TgCyp23. (C) Catalytic effect of TgCyps on protein folding of RNase T1. The increase in fluorescence at 320 nm is shown as a function of the time of refolding in the presence of a fixed concentration of TgCyp18.4 (green), TgCyp23 (blue), and human CypA (red). The control experiment showing the spontaneous refolding of RNase T1 in the absence of Cyps is also displayed (black). (D) Histogram shows the mean value of the exponential folding rate constants *k*_obs_ of RNase T1 in the absence and presence of Cyp variants. Color coding as in C. *n* = 3 independent experiments were performed. The error bars represent standard error from the mean value.

In the chymotrypsin-coupled PPIase assay in the presence of increasing concentrations of AAPF, recombinant TgCyp18.4 and TgCyp23 followed Michaelis–Menten kinetics (Figure 3A,B). The steady-state kinetic parameters are summarized in Table 2. The kinetic characterization of human CypA PPIase activity was included for comparison (Table 2 and Figure S3). Considering Cyps from other organisms, variability in kinetic parameters was observed for the two TgCyps. Indeed, the catalytic efficiency of TgCyp23 for the AAPF peptide substrate was high (*k*_cat_/*K*_m_ = 2.89 × 10^6^ M^–1^ s^–1^) and resembled that of human CypA and some other Cyps,^34−38^ implying that TgCyp23 is a highly active PPIase. On the contrary, TgCyp18.4 is also active as a PPIase but differs significantly from TgCyp23 and human CypA. Indeed, a low level of PPIase activity was observed (*k*_cat_/*K*_m_ = 1.0 × 10^4^ M^–1^ s^–1^) that was only detectable using relatively large amounts of TgCyp18.4, namely, 3 μM compared to 7 nM for TgCyp23 and CypA (see the linear dependency of PPIase activity with increasing enzyme concentration, Figure S4).

**Table 2:** Kinetic Parameters of PPlase Activity in the Presence of the Chromogenic Substrate AAPF

protein	*k*_cat_ (s^–1^)	*K*_m_ (μM)	*k*_cat_/*K*_m_(M^–1^ s^–1^)
CypA	3292 ± 588	661 ± 93	(4.9 ± 0.2) × 10^6^
TgCyp18.4	2.8 ± 0.4	286 ± 39	(1.0 ± 0.2) × 10^4^
TgCyp18.4 H111W	8.31 ± 0.5	719 ± 132	(1.2 ± 0.2) × 10^4^
TgCyp23	2010 ± 381	530 ± 97	(3.8 ± 0.2) × 10^6^

In addition to the PPIase activity, some Cyps can also accelerate the re/folding of RNase T1.^8,39^ We measured the activity of TgCyps in the RNAse T1 refolding assay by recording its tryptophan (Trp59) fluorescence increase at 320 nm. RNase T1 was unfolded in 8 M urea, and its refolding rate was determined by dilution in the absence or presence of TgCyps. As shown in Figure 3C, the refolding rate is slow in the absence of Cyps. However, a marked acceleration of the RNase T1 refolding rate was clearly seen in the presence of human CypA, which was used as the control protein. The refolding of RNase T1 was found to be accelerated by both TgCyp18.4 and TgCyp23, which enhance RNase T1 fluorescence emission compared with the spontaneous process (Figure 3C,D). However, the kinetics of refolding in the presence of TgCyp18.4 and TgCyp23 was slower compared to CypA, with TgCyp18.4 showing a consistent lack of activity relative to human CypA.

### TgCyps Have Different Sensitivities to CsA

**Figure 4:**
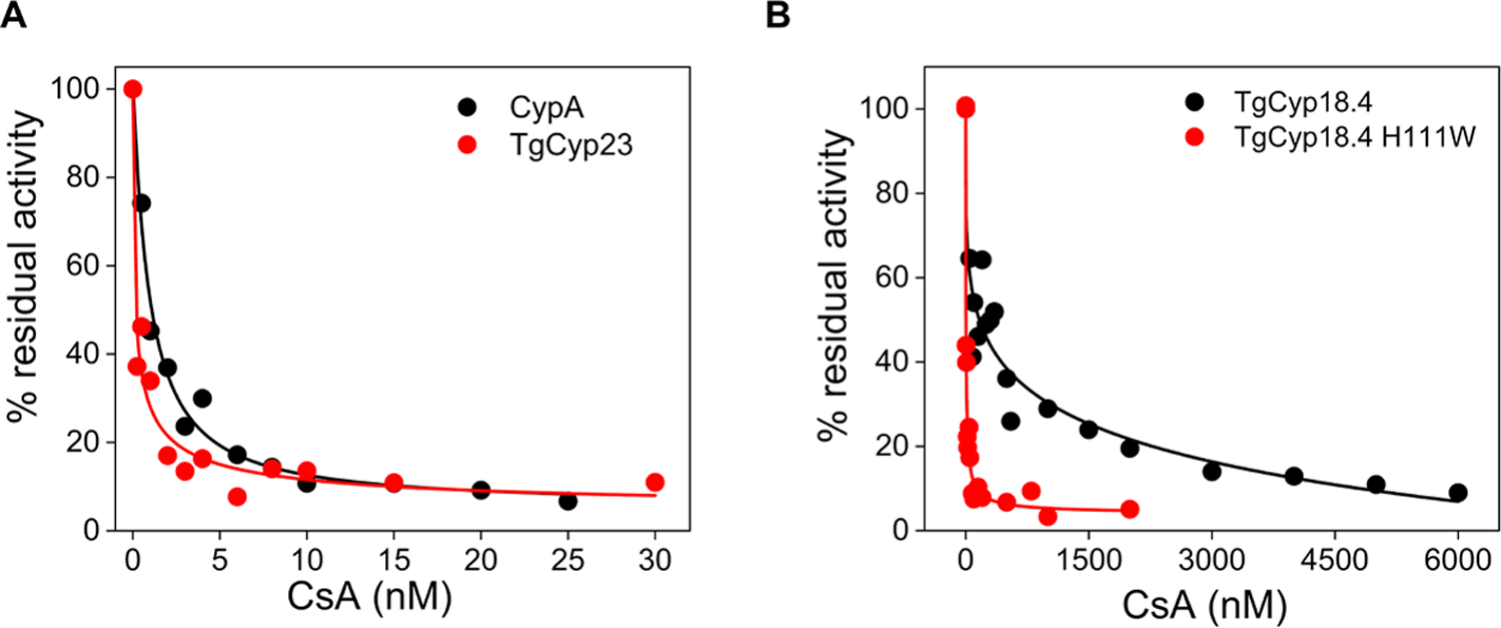
CsA inhibition of recombinant Cyps. (A) PPIase activity of 7 nM TgCyp23 (red) and 7 nM human CypA (black) measured at increasing concentrations of CsA, ranging from 0 to 30 nM. (B) PPIase activity of 3 μM TgCyp18.4 (black) and 3 μM TgCyp18.4 H111W (red) in the presence of increasing amounts of CsA, ranging from 0 to 6 μM. The concentration of AAPF used in the assays was fixed to 100 μM, corresponding to a cis substrate concentration of 40 μM, which is much smaller than the calculated *K*_m_. Therefore, no deviations from the expected first-order kinetics were observed in the presence of CsA.^1^

We next investigated the sensitivity of TgCyp18.4 and TgCyp23 PPIase activity to CsA, which is known to form very tight stoichiometric complexes with CypA.^40^ We found that human CypA PPIase activity, at an enzyme concentration of 7 nM, is inhibited by CsA with an IC_50_ (concentration giving 50% inhibition) of 1 ± 0.3 nM (Figure 4A), in agreement with values previously reported.^1,36,41^ Notably, the IC_50_ of CsA on TgCyp23 was 0.6 ± 0.1 nM and thus very similar to human CypA (Figure 4A), while TgCyp18.4 was 288 times less sensitive to CsA than human CypA (IC_50_ = 288 ± 25 nM) (Figure 4B), being thus more similar to human CyP40 and *Leishmania donovani* Cyp.^28,42^

The weaker inhibitory effect of CsA on TgCyp18.4 could be due to a histidine that substitutes a tryptophan residue, which is essential for the interaction with CsA, as observed for human CyP40. The replacement of His111 by tryptophan through site-directed mutagenesis resulted in a mutant protein with an ∼54-fold higher affinity for CsA (IC_50_ = 5.3 ± 0.2 nM) compared to wild-type TgCyp18.4 (Figure 4B). Interestingly, the mutation did not significantly influence the PPIase activity of the enzyme (Table 2), confirming the essential role of the Trp for the TgCyp18.4–CsA interaction.

To measure the CsA affinity, we next carried out isothermal titration calorimetry (ITC) experiments. Representative isotherms are shown in Figure S5A,B, and optimal thermodynamic parameters are summarized in Table 3. Binding of CsA to both TgCyp23 and TgCyp18.4 was exothermic and showed a single event, consistent with a 1:1 CsA/protein stoichiometry. The CsA affinity for TgCyp23 and human CypA was similar (apparent *K*_d_ values of 14 ± 3 and 82 ± 15 nM, respectively), while that for TgCyp18 was significantly lower (*K*_d_ = 9.3 ± 0.5 μM) (Table 3).

**Table 3:** Thermodynamic Parameters of the Interaction of Cyps with CsA at 20 °C^a^

	*K*_d_ (nM)	Δ*H*(kJ mol^–1^)	Δ*S*(kJ mol^–1^)
CypA + CsA	14 ± 3	–10 658 ± 332	–0.13 ± 0.6
TgCyp23 + CsA	82 ± 15	–17 487 ± 5251	–10.9 ± 2.9
TgCyp18.4 + CsA	9346 ± 530	–1872 ± 873	18.2 ± 2.5

a The reported parameters are the mean ± standard error of the mean (SEM) of at least three independent titrations using two different protein preparations.

We further tested whether the binding of CsA affects the thermal stability of TgCyps using DSC. The DSC traces are shown in Figure S5C,D. Denaturation of both TgCyp18.4 and TgCyp23 indicated the presence of only one main peak with a *T*_m_ of 40 ± 0.2 and 50 ± 0.1 °C, respectively. Interestingly, the addition of CsA did not significantly affect the thermal stability of TgCyp18.4, while a 4 °C increase in *T*_m_ was observed for TgCyp23 in the presence of CsA (Table 1). Consistent with the DSC experiments, the CD thermal denaturation profiles showed no significant effects of CsA on the TgCyp18.4 secondary structure. At the same time, TgCyp23 exhibits a 3 °C increase in *T*_m_ in the presence of CsA (Table 1).

### Three-Dimensional Structure of the TgCyp23:CsA Complex

#### TgCyp23 Overall Structure

**Figure 5:**
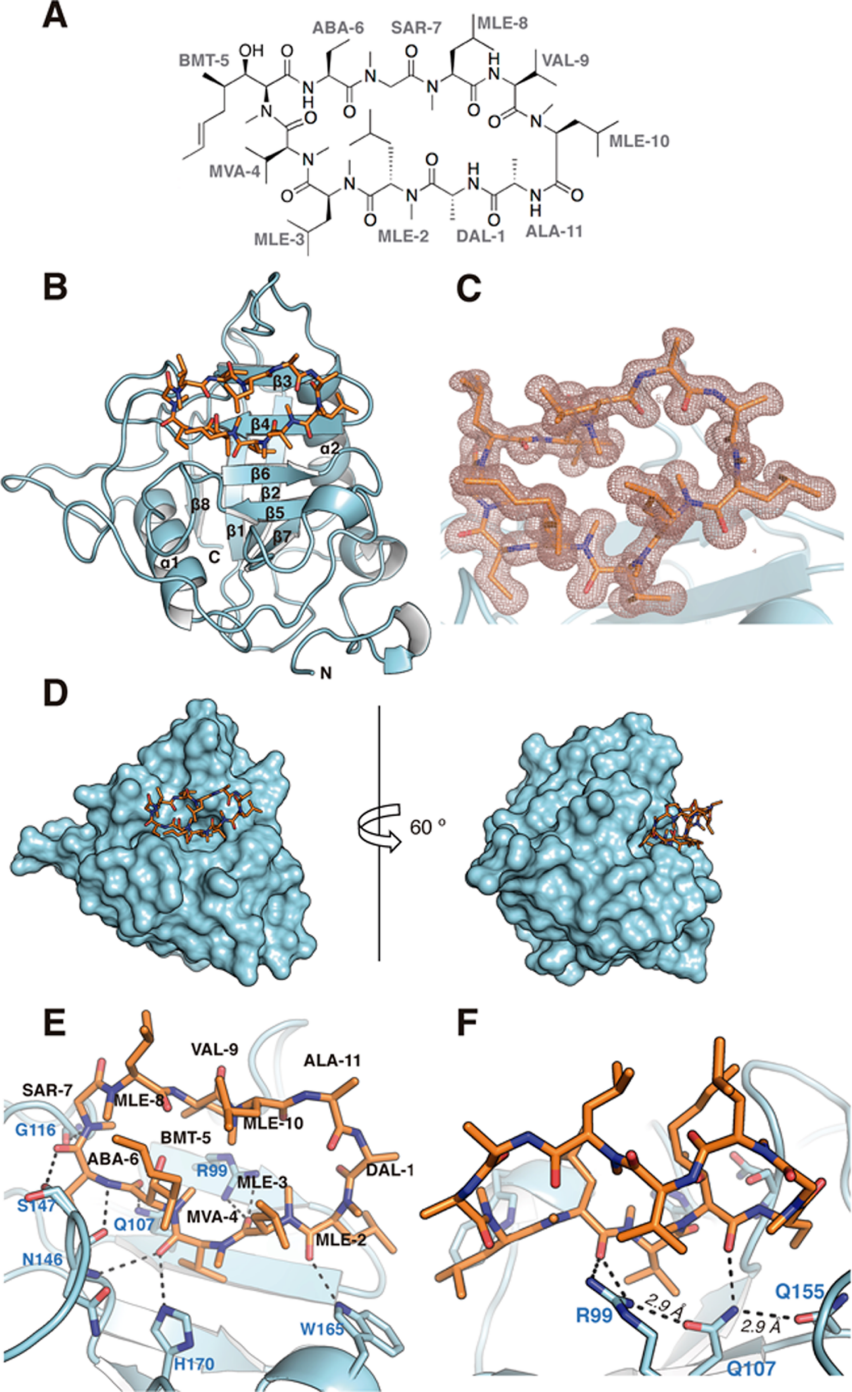
Three-dimensional structure of TgCyp23 in complex with CsA. (A) Chemical structure of CsA. (B) Overall structure of the TgCyp23:CsA complex. TgCyp23 is displayed in blue cartoons, and CsA is depicted as orange sticks. N indicates the N-terminal region. (C) 2*F*_o_ – *F*_c_ electron density map observed for CsA. Map contoured at 1σ. (D) Representation of the molecular surface of TgCyp23 (colored in blue) showing the CsA-binding pocket (CsA depicted as capped sticks). (E) CsA stabilization by TgCyp23. Relevant residues implicated in the interaction are labeled and shown as sticks. (F) Interaction network between nearby protein amino acids (distance in angstroms are indicated). Dashed lines indicate polar interactions.

The crystal structure of TgCyp23 in complex with CsA (Figure 5A) was solved at 1.1 Å resolution by the molecular replacement method using the AlphaFold2^43^-predicted three-dimensional (3D) structure as a search model (see the Methods section and Table S2). There are two monomers in the asymmetric unit with an almost identical structure (RMSD of 0.109 Å for the Cα superposition). The overall structure of TgCyp23 follows the folding observed in the Cyps family and is composed of a β-barrel of eight antiparallel β-strands with two α-helices on the bottom and top of the barrel, all connected by loops (Figure 5B). The high-quality electron density maps observed for TgCyp23 provided information for almost the entire sequence. Additionally, the map showed an electron density unequivocally for the CsA ligand attached to each independent monomer in the crystal (Figure 5C).

A search of structural homologues of TgCyp23 with DALI^44^ revealed human U4/U6 snRNP-specific Cyp (PDB code 1MZW, RMSD of 0.39 Å for 143 superimposed α-carbons) and Cyp from *Plasmodium yoelii* (PDB code 1Z81, RMSD of 0.60 Å for 157 superimposed α-carbons) as the closest homologues. As observed (Figure S6), the Cyp folding is preserved, with main differences in TgCyp23 related to the 40 extra residues in the N-terminal region. Also, structural comparison with the well-characterized human CypA (2CPL PDB code^45^) (Figure S7) reveals conservation of the central structural core (RMSD 0.509 Å for 136 superposed α-carbons).

#### Recognition of CsA by TgCyp23

The complex CsA ligand is located in a pocket built by β3 and β4 and the loops Lβ5β6 (connecting β5 → β6) and Lβ6β7 (connecting β6 → β7), as shown in Figure 5B. The residues constituting the binding pocket are Arg99, Phe104, Met105, Gln106, Ala145, Asn146, Ser147, Gln155, Phe157, Trp165, Leu166, and His170. Structural comparison with CsCyp (*Citrus sinensis* Cyp, PDB code 4JJM) in complex with CsA^46^ shows a similar structure (RMSD of 0.46 Å for 127 superimposed α-carbons) and CsA being located in the same position and with a similar disposition of its residues (Figure S8).

Six of the eleven residues of CsA (Figure 5A) are implicated in contact with the protein (Figure 5E). Stabilization of CsA is performed mainly by polar interactions and van der Waals forces. Analysis with the PISA server^47^ allowed us to find eight hydrogen bonds between CsA and side chains of TgCyp23 and between the O backbone atom of Gly116 and *N*-methylglycine (SAR-7) (Table 4).

**Table 4:** Polar Contacts between TgCyp23 and CsA

protein residues/atoms (chain B)		CsA residues/atoms (chain D)		distance (Å)
Arg99	NH1	MLE-3	O	2.9
Arg99	NH2	MLE-3	O	2.9
Gln107	NE-2	BMT-5	O	3.1
Gly116	O	SAR-7	N	3.1
Ser147	OG	ABA-6	O	2.9
Asn146	O	ABA-6	N	3.0
Asn146	N	MVA-4	O	3.5
Trp165	NE1	MLE-2	O	2.9
His170	NE2	MVA-4	O	3.4

Trp165 residue, located in a 3_10_-helix in the Lβ6β7 loop, is crucial in CsA stabilization due to the formation of a hydrogen bond between its NE1 and the carbonyl O atom of MLE-2 in CsA. Gln107 plays a very important role in the CsA packaging since it both establishes a H-bond interaction with CsA and acts as a bridging residue in a H-bond network that stabilizes the proper orientation of nearby residues Arg99 and Gln155 (Figure 5F).

The interaction pattern observed in TgCyp23 with CsA is conserved in the *C. sinensis* homologue (Figure S8). Notably, the replacement of Ser147 by Ala in the *C. sinensis* homologue (Ala110) entails one less polar interaction, in agreement with the higher affinity observed for TgCyp23 to CsA.

### Predicted 3D Structure of TgCyp18.4

**Figure 6:**
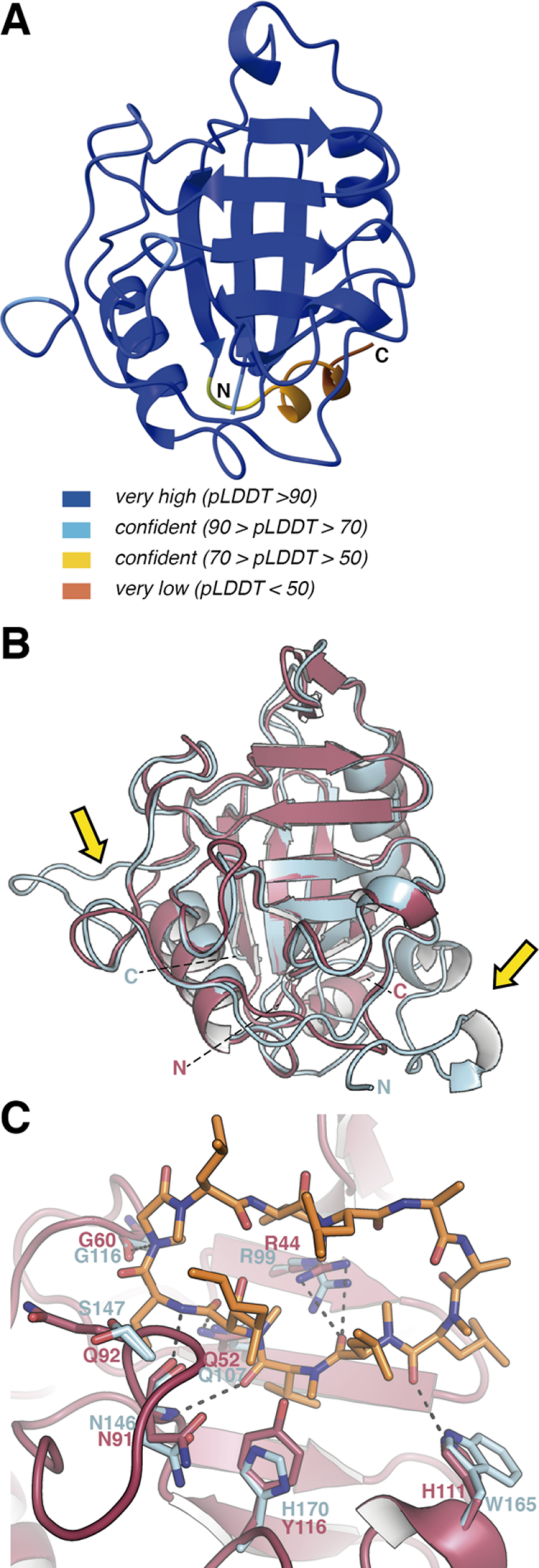
AlphaFold-predicted model for TgCyp18.4. (A) Predicted structure for TgCyp18.4 with colors indicating the reliability of the model. (B) Structural superimposition of the predicted structure of TgCyp18.4 (salmon) onto the crystal structure of TgCyp23 (blue). C and N show C-terminus and N-terminus, respectively. Yellow arrows indicate regions showing larger differences between TgCyp23 and TgCyp18.4. (C) Comparison of CsA-binding pockets in TgCyp23 and TgCyp18.4. TgCyp23 amino acids are displayed in blue sticks, and TgCyp18.4 residues are displayed in red. Dashed lines indicate predicted polar interactions for the TgCyp18.4:CsA complex.

AlphaFold2^43^ predicts a reliable structural model for TgCyp18.4 (Figure 6A). Superimposition of the TgCyp18.4 predicted structure onto the crystal structure of TgCyp23 shows an RMSD of 0.667 Å for 157 superimposed α-carbons with a conserved Cyp core as in TgCyp23 (Figure 6B). The main differences between both Cyps are concentrated at the N- and C-terminal regions. In addition, in TgCyp23, there is a larger region in the Lα1β3 (connecting α1 → β3) that is different between both structures (Figure 6B).

Comparison of the CsA-binding site observed in the crystal structure of the TgCyp23:CsA complex with the putative binding site in TgCyp18.4 (Figure 6C) reveals that some relevant residues remain in the binding pocket like Arg44, Asn91, Gly60, and Gln52 that would conserve the polar interactions with CsA. On the other hand, some differences are also observed in TgCyp18.4. Thus, TgCyp18.4 contains His111 (Trp165 in TgCyp23), Tyr116 (His170 in TgCyp23), and Gln92 (Ser147 in TgCyp23) that could alter the H-bond network observed in TgCyp23.

## Discussion

Identifying and characterizing CsA-binding proteins is crucial in developing potential anti-*Toxoplasma* drugs based on CsA, which was found to possess antiparasitic activity against various apicomplexa.^23,24^ Toward this goal, herein, we have characterized two Cyps from *T. gondii*, TgCyp18.4 and TgCyp23, which were selected among the 13 different Cyps within the genome of *T. gondii* with sequence homology to human CypA. From the alignment of protein sequences, a 40-amino acid N-terminal extension of an unknown function was identified in TgCyp23. Of note, the cyclophilin-like domain is highly conserved in TgCyp23, and TgCyp23 is a highly active PPIase toward the AAPF substrate, with a *k*_cat_/*K*_m_ of 3.8 × 10^6^ M^–1^ s^–1^. This value is very similar to mammalian CypA^28,33,34,37^ and is in accordance with the high degree of conservation of the residues interacting with the substrate in the crystal structure of human CypA.^48^ Moreover, TgCyp23 has a high CsA affinity (*K*_d_ of 82 nM), and its PPIase activity is potently inhibited by CsA with an IC_50_ of 0.6 nM.^1^ Considering this, TgCyp23 is correctly termed a Cyp based on the formal definition as a CsA-binding protein.

On the other hand, TgCyp18.4 has no N-terminal extension but does possess crucial amino acid substitutions in the cyclophilin-like domain in both the active site and CsA-binding region. Compared to mammalian CypA and TgCyp23, TgCyp18.4 has significantly lower PPIase activity with a *k*_cat_/*K*_m_ of 1.0 × 10^4^ M^–1^ s^–1^. Of note, various values of PPIase activity have been described among the Cyp family. For example, there is a 147-fold difference between CypJ and the PPIase activity of human CypA^49^ and a 432-fold difference between Cyp10 and Cyp6 in *C. elegans*.^11^ Similarly, the IC_50_ for CsA against TgCyp18.4 and the affinity of the CsA-TgCyp18.4 interaction were lower (IC_50_ ≈ 288 nM, *K*_d_ ≈ 9.3 μM) compared to the values obtained for TgCyp23, which could be explained by the difference in the CsA–Cyp binding site. Our structural analysis shows that, in TgCyp18.4, a histidine residue replaces the Trp at position 121 (numbered according to human CypA), which is considered an essential CsA binding residue. This histidine, although permissive to CsA binding, could be responsible for the low sensitivity to CsA under our experimental conditions. It has been shown that mutating Trp121 in human CypA to phenylalanine or alanine decreases CsA affinity.^41,50,51^ Replacement of the histidine to a tryptophan in human CypD significantly increases CsA affinity, resulting in a *K*_d_^app^ for CsA of 12 nM and changing the IC_50_ from 1.9 μM to 90 nM.^52^ As expected, mutation of His111 to Trp in TgCyp18.4 led to a mutant that is about 50 times more sensitive to CsA, a result comparable to the sensitivity of human CypA, demonstrating the critical role of this residue for the TgCyp18.4–CsA interaction. On the other hand, the mutation does not affect the PPIase activity; therefore, there appears to be flexibility in the active site for the Trp111 position, and both tryptophan and histidine residues allow enzyme activity.

Of note, in addition to PPIase activity, Cyps were previously described to serve as foldases, contributing to the processes of protein folding. We found that both TgCyps accelerate the refolding of RNase T1 in comparison with the spontaneous processes, suggesting a folding activity for both proteins, albeit lower than for human CypA. Even if the PPIase activity may be the main conserved role of Cyps, which have variable kinetic parameters and sensitivity to CsA, their chaperone activity may be an additional function that is not present in all Cyps. The cis/trans-isomerization of proline residues and molecular chaperone activity could be disconnected, as already suggested for CypA.^6^

The crystal structure of the TgCyp23:CsA complex reveals an overall conservation of Cyp folding and provides atomic information on interactions with CsA, explaining the experimental CsA binding parameters.

The highly confident three-dimensional model of TgCyp18.4 predicted by AlphaFold2 reveals a very highly conserved core with TgCyp23. Structural comparison between CsA recognition in TgCyp23 and TgCyp18.4 shows some differences in the CsA-binding pocket, and the change of some critical residues could explain the lower CsA binding values observed for TgCyp18.4.

**Figure 7:**
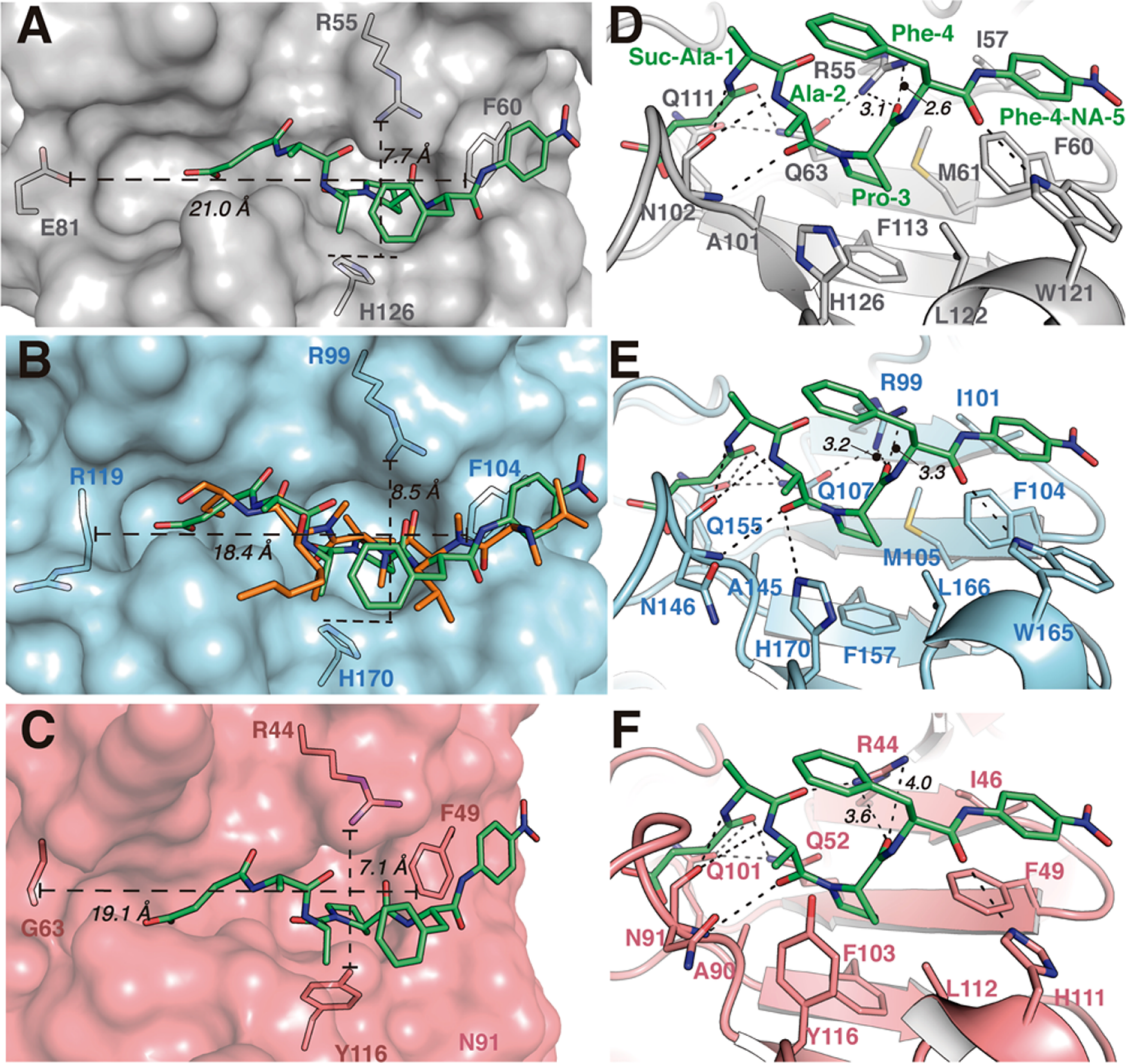
Structural comparison of substrate versus CsA recognition in CypA and *Toxoplasma* Cyps. (A) Detailed view of the binding site in the CypA:AAPF complex. All distances are indicated in dashed lines between the shown atoms in the extremes. (B) TgCyp23 cavity is displayed as the blue surface. AAPF in green sticks and superposed with half-CsA chains (residues 2–6) in orange sticks. (C) TgCyp18.4 surface in salmon and AAPF superposed is depicted as green sticks. (D) CypA:AAPF interaction network (polar contacts are indicated with dotted lines). (E) TgCyp23:AAPF predicted interaction network. (F) TgCyp18.4:AAPF predicted interaction network.

Concerning PPIase activity, structural comparison of our *T. gondii* Cyps with the human CypA in complex with a polypeptide chain (PDB code 1RMH)^48^ provides some interesting clues. In the human CypA structure, the polypeptide AAPF (Figure 7A) is located exactly in the same CsA-binding pocket of TgCyp23 (Figure 7B). The interaction pattern observed for substrate recognition in human CypA (Figure 7D) involves a hydrophobic pocket (residues Ile57, Phe60, Met61, Ala101, Phe113, Leu122), in which the ring of the proline residue to be isomerized is inserted allowing close interactions with the catalytic arginine residue (Arg55). In addition, the backbone of the remaining polypeptide substrate is further stabilized through a network of H-bond interactions (Figure 7D). Interestingly, this pattern is preserved in TgCyp23, and the same polypeptide substrate nicely accommodates into its active site by the direct superimposition of CypA onto TgCyp23 (Figure 7E). Both the hydrophobic pocket (Ile101, Phe104, Met105, Ala145, Phe157, Leu166) and the catalytic arginine (Arg99) are conserved as well as residues in the H-bond network (Figure 7E), providing a substrate-binding site with similar shape and dimensions (Figure 7A,B). It is worth mentioning that a cyclic CsA inhibitor nicely mimics the interaction pattern with a natural substrate as described below (Figure 7B). Thus, the N and O atoms of the AAPF substrate backbone are replaced by similar groups in CsA and the proline residue is replaced by the MVA residue in CsA.

Superpositions of the peptide AAPF into the predicted TgCyp18.4 structure (Figure 7C) provide information about potential interactions (Figure 7F). Cavity dimensions are essential to accommodate the substrate properly. The structural comparison reveals that, while the cavity length is very similar in the three proteins (CypA, TgCyp23, TgCyp18.4), a shortening of the width is observed for TgCyp18.4, a difference mainly due to the replacement of His170 in TgCyp23 by the Tyr116 in TgCyp18.4. This change could affect the entrance of the substrate in TgCyp23 vs TgCyp18.4, in agreement with the observed differences in the experimental PPIase activity for both Cyps.

Further analysis of the interactions also indicates that, while the catalytic arginine has the same orientation in the three structures, it does not have the same distance with the substrate (Figure 7D–F). Shorter distances are observed in the CypA complex (3.1 and 2.6 Å between arginine nitrogen atoms and carboxylic oxygen of the peptide, respectively) compared with TgCyp23 (3.2 and 3.3 Å, respectively) and TgCyp18.4 (3.6 and 4.0 Å, respectively), thus indicating that experimental PPIase activity in TgCyps would require minor rearrangements of the catalytic residue.

The biochemical and structural data presented herein represents a relevant step toward understanding the molecular mechanisms of the anti-*Toxoplasma* action of CsA and may be instrumental in the rational design of new therapeutic drugs modulating TgCyp activity.

## Methods

### Production of Recombinant Proteins

Synthetic genes corresponding to the complete cDNA sequences of TgCyp289260 (TgCyp18.4) and TgCyp285760 (TgCyp23) were purchased from GenScript and cloned into pET28a vector coding for a TEV cleavage site and a His_6_ tag at the N-terminus of the protein sequence. The synthetic gene coding for CypA was a kind gift of Dr. Javier Oroz (Instituto Química-Física Rocasolano, Spain). TgCyp18.4 H111W mutant was produced by site-specific mutagenesis on the pET28a-TgCyp18.4 construct, using the QuikChange site-directed mutagenesis kit (Agilent Technologies).

Heterologous expression of recombinant proteins was performed in BL21 *E. coli* cells following induction with 0.5 mM isopropyl-β-d-1-thiogalactopyranoside (IPTG) for 16 h at 22 °C. Cells were isolated by centrifugation and resuspended in 20 mM Tris–HCl, pH 7.5; 0.5 M NaCl; and 10 mM imidazole, in the presence of EDTA-free protease inhibitor cocktail, DNase I and lysozyme (0.2 μg mL^–1^). Cells were then lysed by sonication on ice and centrifugated to remove membrane debrides. The filtered supernatant was loaded to a Ni-Sepharose column (GE Healthcare) equilibrated with 20 mM Tris–HCl, pH 7.5; 0.5 M NaCl; 10 mM imidazole; and 1 mM DTT and eluted with the same buffer containing 500 mM imidazole. The removal of the His-tag was performed by incubating the proteins for 16 h at 4 °C with a previously prepared recombinant His-tagged TEV-protease (ratio 1:100), reloading the tag-free proteins into a Ni-Sepharose column and collecting them in the flow-through. Eluted proteins were concentrated using Vivaspin concentrators (Sartorius), and the purity of the enzymes was checked by sodium dodecyl sulfate poly(acrylamide) gel electrophoresis (SDS-PAGE) to be >95%.

### Analytical Gel Filtration

The apparent molecular weight and the oligomeric state of TgCyp variants were analyzed by analytical gel filtration. Protein samples (2 mg mL^–1^) were loaded onto a Superdex 75 Increase 10/300 GL column (GE Healthcare) in 20 mM Tris–HCl, pH 7.5; 150 mM NaCl; and 1 mM DTT. A calibration curve was created, following protocols in refs (53) and (54).

### Determination of PPIase Activity and Inhibitor Studies

PPIase activity was determined using the synthetic peptide *N*-succinyl-Ala-Ala-Pro-Phe-*p*-nitroanilide (AAPF) based on the protease-coupled assay as described in ref (33). The assay was performed in 50 mM Hepes, pH 8, and 100 mM NaCl at 0 °C. Briefly, unless differently indicated in the text, 7 nM Cyps were mixed with increasing substrate concentrations (75–4000 μM AAPF solubilized in 470 mM LiCl, 100% 2,2,2-trifluorethanol (TFE)) at a final volume of 200 μL in a 1-cm path cuvette. Once the temperature reached 0 °C, chymotrypsin solution, dissolved in HCl 1 mM, was added at 1.8 mg mL^–1^ to start the reaction.

Based on the concentration of the substrate, changes in absorbance were recorded at 390, 445, 450, and 460 nm. The product was quantified using the extinction molar coefficients 13 300 M^–1^ cm^–1^ (390 nm), 1250 M^–1^ cm^–1^ (445 nm), 995 M^–1^ cm^–1^ (450 nm), and 267 M^–1^ cm^–1^ (460 nm). The TFE and LiCl concentrations used in the assay did not exceed 2.5% (v/v) and 12 mM, respectively. The initial cis concentration of AAPF was measured by dissolving the substrate to a final concentration of 100 mM in the presence of chymotrypsin (2 mg mL^–1^) and recording the absorbance at 390 nm. This value corresponds to the trans-isomer present in the initial AAPF solution. Successively, Cyp was added in large excess to enhance the cis-to-trans isomerization. The cis population was estimated by the difference between the final and the initial absorbance. In our experimental conditions, the cis conformer represented the 40–60% of the total isomer present in the original substrate solution.

Inhibition assays were performed by incubating the enzymes for 10 min on ice with increasing amounts of CsA ranging from 0 to 6000 nM before the addition of the AAPF peptide.

For all of the experiments, the slope of the catalyzed reaction was corrected for the spontaneous thermal cis–trans isomerization in the presence of the only chymotrypsin.

All measurements were repeated as triplicate, and the error was calculated as the mean standard error of the three measurements. The error associated with the parameter *k*_cat_/*K*_m_ was propagated in agreement with the following equation eq 1
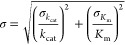
1

### Protein Folding Assay

A sample of 50 μM RNase T1 (Thermo Fisher) was unfolded by incubation in 8 M urea for 2 h at 5 °C in 100 mM Tris–HCl, pH 8. Refolding of RNase T1 was started by diluting it with the same buffer without urea, in the absence or presence of Cyp at a final RNAse T1/Cyp ratio 1:1, to a final RNase T1 concentration of 250 nM. The refolding of RNase T1 was monitored by tryptophan fluorescence (excitation wavelength at 280 nm) recording the emission at 320 nm for 30 min at 5 °C using a Cary Eclipse fluorescence spectrophotometer with constant mixing of the solution. The pseudo-first-order rate constant *k*_obs_ was calculated using the following equation
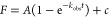
2where *F* is the fluorescence at 320 nm, *t* is the time, *A* is the burst amplitude, and *c* is the end point.

All measurements were repeated as triplicate, and the error was calculated as the mean standard error of the three measurements.

### Circular Dichroism Spectroscopy

The secondary structure of the proteins was analyzed by circular dichroism (CD) spectroscopy in the far-UV region (190–260 nm) on a Jasco J-1500 spectropolarimeter with a Peltier temperature controller, following the protocols described in refs (55) and (56). Briefly, far-UV spectra of ∼0.2 mg mL^–1^ proteins were collected at 25 °C in 5 mM Hepes, pH 8.0, and 10 mM NaCl, and each recorded spectrum was the average of three accumulations. The percentage of the secondary structure content of TgCyp18.4 and TgCyp23 was estimated using DichroWeb.^57^

Denaturation profiles, in the absence or presence of equimolar concentrations of CsA, were recorded by monitoring the CD signal at 222 nm in a temperature window from 15 to 100 °C (scan rate 1.5 °C min^–1^).

### Differential Scanning Calorimetry (DSC)

DSC experiments were conducted using a nano-DSC (TA instrument) with a cell volume of 300 μL.^58^ Protein samples, containing 60–80 μM Cyp in 5 mM Hepes, pH 8.0; 10 mM NaCl; and 0.2% ethanol, in the presence or absence of CsA, were heated from 15 to 100 °C at a scan rate of 60 °C h^–1^. Data were fitted according to a two-state model using NanoAnalyze software (TA instrument).

### Isothermal Titration Calorimetry

ITC experiments were performed on a VP-ITC instrument from MicroCal (Northampton, MA) as described elsewhere.^59,60^ CsA at 8–20 μM was titrated with 2–3 μL of 66–400 μM Cyp solution at 20 °C (13–19 total injections) in the ITC measuring buffer (50 mM Hepes, pH 8.0; 100 mM NaCl) supplemented with 0.4% ethanol (v/v) keeping a time gap of 150 s between injections. All buffers were filtered (0.22 μm) and degassed immediately before use. A reference injection of protein into buffer without CsA was performed, and the reference was subtracted from each experiment. Three independent repetitions were made for each titration set. Data were fitted by one set of sites using MicroCal Origin. The fitting models were used to obtain apparent dissociation constants *K*_d_, enthalpy changes (Δ*H*), and entropy changes (Δ*S*). For the fitting of TgCyp18.4 experiments, the number of sites (*N*) was set equal to 1.

### NMR Experiments

^1^H monodimensional (1D) NMR experiments of samples of TgCyp18.4 and TgCyp23 at a concentration of 70 and 100 μM, respectively, were recorded on a Bruker AVANCE III spectrometer operating at a 600 MHz proton Larmor frequency, equipped with a triple resonance Prodigy CryoProbe. In total, 65 536 complex points were recorded in the proton dimension. All experiments were done at 25 °C in 20 mM Na_2_HPO_4_/NaH_2_PO_4_, pH 7.2; 150 mM NaCl; and 5% D_2_O.

### Crystallization of the TgCyp23:CsA Complex

TgCyp23 was concentrated to 26 mg mL^–1^ using a centricon-10 device. Protein was incubated with CsA in powder in a molar ratio 1:10 and stirred during 3 days at 4 °C. The solution was then filtered through a 0.25 μm filter to remove the excess solid. The crystallization was carried out using a sitting drop vapor-diffusion technique and testing Hampton, Jena Bioscience, Quiagen, and Molecular Dimensions commercial screens with an Oryx crystallization robot. All of the crystallization conditions were tested by mixing 150 nL of the protein solution with 150 nL precipitant solution, equilibrated against 65 μL of solution in the reservoir. Best crystals grew up at 18 °C after 3 days in a precipitant solution that contains 0.1 M citric acid, pH 3.5, and 25% poly(ethylene glycol) 3350.

### Data Collection and Structural Determination of the TgCyp23:CsA Complex

Diffraction data sets were collected in the XALOC beamline at the ALBA synchrotron (Barcelona, Spain) using a Pilatus 6 M detector and a fixed wavelength of 0.97918 Å. Collected images were processed using XDS^61^ and scaled using AIMLESS from the CCP4 package.^62^ Crystals diffracted up to 1.1 Å resolution and belonged to the monoclinic *P*2_1_ space group, with unit cell parameters *a* = 38.40 Å, *b* = 119.42 Å, *c* = 46.35 Å, and α = γ = 90°, and β = 103.62°. Crystals present two monomers in the asymmetric unit with a 44.86% of solvent content. Structure determination was performed by the molecular replacement method with a Phaser MR program from the CCP4 package using the AlphaFold2^38^-predicted 3D structure as a search model.

The model was finally manually completed and subjected to iterative cycles of model building and refinement with Coot,^63^ REFMAC,^64^ PHENIX,^65^ and PDB-REDO.^66^

Refinement also included TLS refinement and individual anisotropic *B*-values. Statistics for the data processing and refinement are summarized in Table S2.

### Alphafold Modeling

TgCyp23 and TgCyp18.4 structural predictions were performed using AlphaFold v2.1 with default parameters. Thus, the prediction was launched with one homooligomer, mmseqs2 option for the multiple sequence alignment (MSA) searching, unpaired mode (that generates separate MSA for each protein), and no filters applied for cov (minimum coverage with query (%)) or qid (minimum sequence identity with query (%)). The generated models obtained applying default settings (five num_models, three max_recycles and use_ptm) were very similar, and the one with the highest rank based on pLDDT was used for further analysis.

## Supporting Information Available

Secondary structure content (%) of TgCyp18.4 and TgCyp23; data collection and refinement statistics for the TgCyp23:CsA complex; multiple sequence alignment of Cyps; SDS-PAGE and SEC of purified proteins; dependence of product formation on the concentration of Cyp enzymes; ITC and DSC profiles of CsA binding to TgCyps; structural comparison among TgCyp23 homologues and with human CypA; 3D structure of the CsCyp:CsA complex (PDF)

## Accession Codes

PDB ID Code: 8B58

## Notes

Authors will release the atomic coordinates and experimental data upon article publication.

## References

[ref1] Fischer G.; Wittmann-Liebold B.; Lang K.; Kiefhaber T.; Schmid F. X. Cyclophilin and peptidyl-prolyl cis-trans isomerase are probably identical proteins. Nature 1989, 337, 476–478. 10.1038/337476a0. 2492638

[ref2] Göthel S. F.; Marahiel M. A. Peptidyl-prolyl cis-trans isomerases, a superfamily of ubiquitous folding catalysts. Cell. Mol. Life Sci. 1999, 55, 423–436. 10.1007/s000180050299. 10228556 PMC11146858

[ref3] Singh H.; Kaur K.; Singh M.; Kaur G.; Singh P. Plant Cyclophilins: Multifaceted Proteins With Versatile Roles. Front. Plant Sci. 2020, 11, 585212 10.3389/fpls.2020.585212. 33193535 PMC7641896

[ref4] Radhakrishnan J.; Bazarek S.; Chandran B.; Gazmuri R. J. Cyclophilin-D: a resident regulator of mitochondrial gene expression. FASEB J. 2015, 29, 2734–2748. 10.1096/fj.14-263855. 25837584

[ref5] Ünal C. M.; Steinert M. Microbial peptidyl-prolyl cis/trans isomerases (PPIases): virulence factors and potential alternative drug targets. Microbiol. Mol. Biol. Rev. 2014, 78, 544–571. 10.1128/MMBR.00015-14. 25184565 PMC4187684

[ref6] Favretto F.; Flores D.; Baker J. D.; Strohäker T.; Andreas L. B.; Blair L. J.; Becker S.; Zweckstetter M. Catalysis of proline isomerization and molecular chaperone activity in a tug-of-war. Nat. Commun. 2020, 11, 6046 10.1038/s41467-020-19844-0. 33247146 PMC7695863

[ref7] Babu M.; Favretto F.; de Opakua A. I.; Rankovic M.; Becker S.; Zweckstetter M. Proline/arginine dipeptide repeat polymers derail protein folding in amyotrophic lateral sclerosis. Nat. Commun. 2021, 12, 3396 10.1038/s41467-021-23691-y. 34099711 PMC8184751

[ref8] Zhang X.-C.; Wang W.-D.; Wang J.-S.; Pan J.-C. PPIase independent chaperone-like function of recombinant human Cyclophilin A during arginine kinase refolding. FEBS Lett. 2013, 587, 666–672. 10.1016/j.febslet.2013.01.028. 23376614

[ref9] Ou W. B.; Luo W.; Park Y. D.; Zhou H. M. Chaperone-like activity of peptidyl-prolyl cis-trans isomerase during creatine kinase refolding. Protein Sci. 2008, 10, 2346–2353. 10.1110/ps.23301. PMC2374073 11604540

[ref10] Davis T. L.; Walker J. R.; Campagna-Slater V.; Finerty P. J. Jr.; Paramanathan R.; Bernstein G.; MacKenzie F.; Tempel W.; Ouyang H.; Lee W. H.; Eisenmesser E. Z.; Dhe-Paganon S. Structural and Biochemical Characterization of the Human Cyclophilin Family of Peptidyl-Prolyl Isomerases. PLoS Biol. 2010, 8, e1000439 10.1371/journal.pbio.1000439. 20676357 PMC2911226

[ref11] Page A. P.; MacNiven K.; Hengartner M. O. Cloning and biochemical characterization of the cyclophilin homologues from the free-living nematode *Caenorhabditis elegans*. Biochem. J. 1996, 317, 179–185. 10.1042/bj3170179. 8694762 PMC1217461

[ref12] Galat A. Peptidylprolyl cis/trans isomerases (immunophilins): biological diversity--targets--functions. Curr. Top. Med. Chem. 2003, 3, 1315–1347. 10.2174/1568026033451862. 12871165

[ref13] Arevalo-Rodriguez M.; Wu X.; Hanes S. D.; Heitman J. Prolyl isomerases in yeast. Front. Biosci. 2004, 9, 2420–2446. 10.2741/1405. 15353296

[ref14] Galat A. Variations of Sequences and Amino Acid Compositions of Proteins That Sustain Their Biological Functions: An Analysis of the Cyclophilin Family of Proteins. Arch. Biochem. Biophys. 1999, 371, 149–162. 10.1006/abbi.1999.1434. 10545201

[ref15] Romano P. G. N.; Horton P.; Gray J. E. The Arabidopsis cyclophilin gene family. Plant Physiol. 2004, 134, 1268–1282. 10.1104/pp.103.022160. 15051864 PMC419803

[ref16] Mainali H. R.; Chapman P.; Dhaubhadel S. Genome-wide analysis of Cyclophilin gene family in soybean (*Glycine max*). BMC Plant Biol. 2014, 14, 282 10.1186/s12870-014-0282-7. 25348509 PMC4220052

[ref17] Hanhart P.; Thieß M.; Amari K.; Bajdzienko K.; Giavalisco P.; Heinlein M.; Kehr J. Bioinformatic and expression analysis of the *Brassica napus* L. cyclophilins. Sci. Rep. 2017, 7, 1514 10.1038/s41598-017-01596-5. 28473712 PMC5431436

[ref18] Ahn J. C.; Kim D. W.; You Y. N.; Seok M. S.; Park J. M.; Hwang H.; Kim B. G.; Luan S.; Park H. S.; Cho H. S. Classification of rice (*Oryza sativa* L. Japonica nipponbare) immunophilins (FKBPs, CYPs) and expression patterns under water stress. BMC Plant Biol. 2010, 10, 253 10.1186/1471-2229-10-253. 21087465 PMC3012604

[ref19] Schiene-Fischer C. Multidomain Peptidyl Prolyl cis/trans Isomerases. Biochim. Biophys. Acta, Gen. Subj. 2015, 1850, 2005–2016. 10.1016/j.bbagen.2014.11.012. 25445709

[ref20] Clipstone N. A.; Crabtree G. R. Identification of calcineurin as a key signalling enzyme in T-lymphocyte activation. Nature 1992, 357, 695–697. 10.1038/357695a0. 1377362

[ref21] Jin L.; Harrison S. C. Crystal structure of human calcineurin complexed with cyclosporin A and human cyclophilin. Proc. Natl. Acad. Sci. U.S.A. 2002, 99, 13522–13526. 10.1073/pnas.212504399. 12357034 PMC129706

[ref22] Bell A.; Monaghan P.; Page A. P. Peptidyl-prolyl cis–trans isomerases (immunophilins) and their roles in parasite biochemistry, host–parasite interaction and antiparasitic drug action. Int. J. Parasitol. 2006, 36, 261–276. 10.1016/j.ijpara.2005.11.003. 16443228

[ref23] Page A. P.; Kumar S.; Carlow C. K. S. Parasite cyclophilins and antiparasite activity of cyclosporin A. Parasitol. Today 1995, 11, 385–388. 10.1016/0169-4758(95)80007-7. 15275401

[ref24] Chappell L. H.; Wastling J. M. Cyclosporin A: antiparasite drug, modulator of the host-parasite relationship and immunosuppressant. Parasitology 1992, 105, S25–S40. 10.1017/S0031182000075338. 1308927

[ref25] Nickell S. P.; Scheibel L. W.; Cole G. A. Inhibition by cyclosporin A of rodent malaria in vivo and human malaria in vitro. Infect. Immun. 1982, 37, 1093–1100. 10.1128/iai.37.3.1093-1100.1982. 6752020 PMC347653

[ref26] Bogdan C.; Streck H.; Röllinghoff M.; Solbach W. Cyclosporin A enhances elimination of intracellular *L. major* parasites by murine macrophages. Clin. Exp. Immunol. 1989, 75, 141. 2702771 PMC1541875

[ref27] Hoerauf A.; Rascher C.; Bang R.; Pahl A.; Solbach W.; Brune K.; Röllinghoff M.; Bang H. Host-cell cyclophilin is important for the intracellular replication of *Leishmania major*. Mol. Microbiol. 1997, 24, 421–429. 10.1046/j.1365-2958.1997.3401716.x. 9159527

[ref28] Yau W.-L.; Blisnick T.; Taly J.-F.; Helmer-Citterich M.; Schiene-Fischer C.; Leclercq O.; Li J.; Schmidt-Arras D.; Morales M. A.; Notredame C.; Romo D.; Bastin P.; Späth G. F. Cyclosporin A Treatment of *Leishmania donovani* Reveals Stage-Specific Functions of Cyclophilins in Parasite Proliferation and Viability. PLoS Neglected Trop. Dis. 2010, 4, e729 10.1371/journal.pntd.0000729. PMC2894131 20614016

[ref29] Krücken J.; Greif G.; von Samson-Himmelstjerna G. In silico analysis of the cyclophilin repertoire of apicomplexan parasites. Parasites Vectors 2009, 2, 27 10.1186/1756-3305-2-27. 19555495 PMC2713222

[ref30] High K. P.; Joiner K. A.; Handschumacher R. E. Isolation, cDNA sequences, and biochemical characterization of the major cyclosporin-binding proteins of *Toxoplasma gondii*. J. Biol. Chem. 1994, 269, 9105–9112. 10.1016/S0021-9258(17)37083-7. 8132648

[ref31] Ibrahim H. M.; Bannai H.; Xuan X.; Nishikawa Y. *Toxoplasma gondii* cyclophilin 18-mediated production of nitric oxide induces Bradyzoite conversion in a CCR5-dependent manner. Infect. Immun. 2009, 77, 3686–3695. 10.1128/IAI.00361-09. 19564392 PMC2737997

[ref32] Aryal S.; Hsu H.-M.; Lou Y.-C.; Chu C.-H.; Tai J.-H.; Hsu C.-H.; Chen C. N-Terminal Segment of TvCyP2 Cyclophilin from *Trichomonas vaginalis* Is Involved in Self-Association, Membrane Interaction, and Subcellular Localization. Biomolecules 2020, 10, 1239 10.3390/biom10091239. 32859063 PMC7563477

[ref33] Kofron J. L.; Kuzmic P.; Kishore V.; Colon-Bonilla E.; Rich D. H. Determination of kinetic constants for peptidyl prolyl cis-trans isomerases by an improved spectrophotometric assay. Biochemistry 1991, 30, 6127–6134. 10.1021/bi00239a007. 2059621

[ref34] Liu J.; Albers M. W.; Chen C. M.; Schreiber S. L.; Walsh C. T. Cloning, expression, and purification of human cyclophilin in *Escherichia coli* and assessment of the catalytic role of cysteines by site-directed mutagenesis. Proc. Natl. Acad. Sci. U.S.A. 1990, 87, 2304–2308. 10.1073/pnas.87.6.2304. 2179953 PMC53675

[ref35] Jakob R. P.; Schmidpeter P. A.; Koch J. R.; Schmid F. X.; Maier T. Structural and Functional Characterization of a Novel Family of Cyclophilins, the AquaCyps. PLoS One 2016, 11, e0157070 10.1371/journal.pone.0157070. 27276069 PMC4898713

[ref36] Berriman M.; Fairlamb A. H. Detailed characterization of a cyclophilin from the human malaria parasite *Plasmodium falciparum*. Biochem. J. 1998, 334, 437–445. 10.1042/bj3340437. 9716503 PMC1219707

[ref37] Yurchenko V.; Xue Z.; Sherry B.; Bukrinsky M. Functional analysis of *Leishmania major* cyclophilin. Int. J. Parasitol. 2008, 38, 633–9. 10.1016/j.ijpara.2007.10.001. 17991468 PMC2377454

[ref38] Bergsma D. J.; Eder C.; Gross M.; Kersten H.; Sylvester D.; Appelbaum E.; Cusimano D.; Livi G. P.; McLaughlin M. M.; Kasyan K. The cyclophilin multigene family of peptidyl-prolyl isomerases. Characterization of three separate human isoforms. J. Biol. Chem. 1991, 266, 23204–23214. 10.1016/S0021-9258(18)54484-7. 1744118

[ref39] Mok D.; Allan R. K.; Carrello A.; Wangoo K.; Walkinshaw M. D.; Ratajczak T. The chaperone function of cyclophilin 40 maps to a cleft between the prolyl isomerase and tetratricopeptide repeat domains. FEBS Lett. 2006, 580, 2761–2768. 10.1016/j.febslet.2006.04.039. 16650407

[ref40] Ke H.; Mayrose D.; Belshaw P. J.; Alberg D. G.; Schreiber S. L.; Chang Z. Y.; Etzkorn F. A.; Ho S.; Walsh C. T. Crystal structures of cyclophilin A complexed with cyclosporin A and N-methyl-4-[(E)-2-butenyl]-4,4-dimethylthreonine cyclosporin A. Structure 1994, 2, 33–44. 10.1016/S0969-2126(00)00006-X. 8075981

[ref41] Zydowsky L. D.; Etzkorn F. A.; Chang H. Y.; Ferguson S. B.; Stolz L. A.; Ho S. I.; Walsh C. T. Active site mutants of human cyclophilin A separate peptidyl-prolyl isomerase activity from cyclosporin A binding and calcineurin inhibition. Protein Sci. 1992, 1, 1092–1099. 10.1002/pro.5560010903. 1338979 PMC2142182

[ref42] Hoffmann K.; Kakalis L. T.; Anderson K. S.; Armitage I. M.; Handschumacher R. E. Expression of human cyclophilin-40 and the effect of the His141-->Trp mutation on catalysis and cyclosporin A binding. Eur. J. Biochem. 1995, 229, 188–193. 10.1111/j.1432-1033.1995.tb20454.x. 7744028

[ref43] Jumper J.; Evans R.; Pritzel A.; Green T.; Figurnov M.; Ronneberger O.; Tunyasuvunakool K.; Bates R.; Žídek A.; Potapenko A.; Bridgland A.; Meyer C.; Kohl S. A. A.; Ballard A. J.; Cowie A.; Romera-Paredes B.; Nikolov S.; Jain R.; Adler J.; Back T.; Petersen S.; Reiman D.; Clancy E.; Zielinski M.; Steinegger M.; Pacholska M.; Berghammer T.; Bodenstein S.; Silver D.; Vinyals O.; Senior A. W.; Kavukcuoglu K.; Kohli P.; Hassabis D. Highly accurate protein structure prediction with AlphaFold. Nature 2021, 596, 583–589. 10.1038/s41586-021-03819-2. 34265844 PMC8371605

[ref44] Holm L. DALI and the persistence of protein shape. Protein Sci. 2020, 29, 128–140. 10.1002/pro.3749. 31606894 PMC6933842

[ref45] Ke H. Similarities and differences between human cyclophilin A and other β-barrel structures: Structural refinement at 1.63 Å resolution. J. Mol. Biol. 1992, 228, 539–550. 10.1016/0022-2836(92)90841-7. 1453463

[ref46] Campos B. M.; Sforça M. L.; Ambrosio A. L.; Domingues M. N.; Brasil de Souza Tde A.; Barbosa J. A.; Paes Leme A. F.; Perez C. A.; Whittaker S. B.; Murakami M. T.; Zeri A. C.; Benedetti C. E. A redox 2-Cys mechanism regulates the catalytic activity of divergent cyclophilins. Plant Physiol. 2013, 162, 1311–1323. 10.1104/pp.113.218339. 23709667 PMC3707534

[ref47] Krissinel E.; Henrick K. Detection of Protein Assemblies in Crystals. In Computational Life Sciences; Springer Berlin Heidelberg: Berlin, Heidelberg, 2005.

[ref48] Zhao Y.; Ke H. Crystal Structure Implies That Cyclophilin Predominantly Catalyzes the Trans to Cis Isomerization. Biochemistry 1996, 35, 7356–7361. 10.1021/bi9602775. 8652511

[ref49] Chen J.; Liefke R.; Jiang L.; Wang J.; Huang C.; Gong Z.; Schiene-Fischer C.; Yu L. Biochemical Features of Recombinant Human Cyclophilin J. Anticancer Res. 2016, 36, 1175–1180. 10.1158/1538-7445.AM2016-1175. 26977013

[ref50] Bossard M. J.; Koser P. L.; Brandt M.; Bergsma D. J.; Levy M. A. A single Trp121 to Ala121 mutation in human cyclophilin alters cyclosporin A affinity and peptidyl-prolyl isomerase activity. Biochem. Biophys. Res. Commun. 1991, 176, 1142–1148. 10.1016/0006-291X(91)90404-U. 2039499

[ref51] Liu J.; Chen C. M.; Walsh C. T. Human and *Escherichia coli* cyclophilins: sensitivity to inhibition by the immunosuppressant cyclosporin A correlates with a specific tryptophan residue. Biochemistry 1991, 30, 2306–2310. 10.1021/bi00223a003. 2001362

[ref52] Kajitani K.; Fujihashi M.; Kobayashi Y.; Shimizu S.; Tsujimoto Y.; Miki K. Crystal structure of human cyclophilin D in complex with its inhibitor, cyclosporin A at 0.96-A resolution. Proteins 2007, 70, 1635–1639. 10.1002/prot.21855. 18076075

[ref53] La Verde V.; Dominici P.; Astegno A. Determination of Hydrodynamic Radius of Proteins by Size Exclusion Chromatography. Bio-Protocol 2017, 7, e2230 10.21769/BioProtoc.2230. 34541230 PMC8410290

[ref54] Astegno A.; Maresi E.; Bertoldi M.; La Verde V.; Paiardini A.; Dominici P. Unique substrate specificity of ornithine aminotransferase from *Toxoplasma gondii*. Biochem. J. 2017, 474, 939–955. 10.1042/BCJ20161021. 28126740

[ref55] Trande M.; Pedretti M.; Bonza M. C.; Di Matteo A.; D’Onofrio M.; Dominici P.; Astegno A. Cation and peptide binding properties of CML7, a calmodulin-like protein from *Arabidopsis thaliana*. J. Inorg. Biochem. 2019, 199, 110796 10.1016/j.jinorgbio.2019.110796. 31419675

[ref56] La Verde V.; Trande M.; D’Onofrio M.; Dominici P.; Astegno A. Binding of calcium and target peptide to calmodulin-like protein CML19, the centrin 2 of *Arabidopsis thaliana*. Int. J. Biol. Macromol. 2018, 108, 1289–1299. 10.1016/j.ijbiomac.2017.11.044. 29129631

[ref57] Miles A. J.; Ramalli S. G.; Wallace B. A. DichroWeb, a website for calculating protein secondary structure from circular dichroism spectroscopic data. Protein Sci. 2022, 31, 37–46. 10.1002/pro.4153. 34216059 PMC8740839

[ref58] Conter C.; Fruncillo S.; Favretto F.; Fernández-Rodríguez C.; Dominici P.; Martínez-Cruz L. A.; Astegno A. Insights into Domain Organization and Regulatory Mechanism of Cystathionine Beta-Synthase from *Toxoplasma gondii*. Int. J. Mol. Sci. 2022, 23, 8169 10.3390/ijms23158169. 35897745 PMC9331509

[ref59] Bombardi L.; Favretto F.; Pedretti M.; Conter C.; Dominici P.; Astegno A. Conformational Plasticity of Centrin 1 from *Toxoplasma gondii* in Binding to the Centrosomal Protein SFI1. Biomolecules 2022, 12, 1115 10.3390/biom12081115. 36009009 PMC9406199

[ref60] Conter C.; Bombardi L.; Pedretti M.; Favretto F.; Di Matteo A.; Dominici P.; Astegno A. The interplay of self-assembly and target binding in centrin 1 from *Toxoplasma gondii*. Biochem. J. 2021, 478, 2571–2587. 10.1042/BCJ20210295. 34114596 PMC8286830

[ref61] Kabsch W. XDS. Acta Crystallogr., Sect. D: Biol. Crystallogr. 2010, 66, 125–132. 10.1107/S0907444909047337. 20124692 PMC2815665

[ref62] Winn M. D.; Ballard C. C.; Cowtan K. D.; Dodson E. J.; Emsley P.; Evans P. R.; Keegan R. M.; Krissinel E. B.; Leslie A. G.; McCoy A.; McNicholas S. J.; Murshudov G. N.; Pannu N. S.; Potterton E. A.; Powell H. R.; Read R. J.; Vagin A.; Wilson K. S. Overview of the CCP4 suite and current developments. Acta Crystallogr., Sect. D: Biol. Crystallogr. 2011, 67, 235–242. 10.1107/S0907444910045749. 21460441 PMC3069738

[ref63] Emsley P.; Lohkamp B.; Scott W. G.; Cowtan K. Features and development of Coot. Acta Crystallogr., Sect. D: Biol. Crystallogr. 2010, 66, 486–501. 10.1107/S0907444910007493. 20383002 PMC2852313

[ref64] Murshudov G. N.; Skubák P.; Lebedev A. A.; Pannu N. S.; Steiner R. A.; Nicholls R. A.; Winn M. D.; Long F.; Vagin A. A. REFMAC5 for the refinement of macromolecular crystal structures. Acta Crystallogr., Sect. D: Biol. Crystallogr. 2011, 67, 355–367. 10.1107/S0907444911001314. 21460454 PMC3069751

[ref65] Afonine P. V.; Grosse-Kunstleve R. W.; Echols N.; Headd J. J.; Moriarty N. W.; Mustyakimov M.; Terwilliger T. C.; Urzhumtsev A.; Zwart P. H.; Adams P. D. Towards automated crystallographic structure refinement with phenix.refine. Acta Crystallogr., Sect. D: Biol. Crystallogr. 2012, 68, 352–367. 10.1107/S0907444912001308. 22505256 PMC3322595

[ref66] Joosten R. P.; Long F.; Murshudov G. N.; Perrakis A. The PDB_REDO server for macromolecular structure model optimization. IUCrJ 2014, 1, 213–220. 10.1107/S2052252514009324. PMC4107921 25075342

