# Supplementary material for: Structural Basis for Cyclosporin Isoform-Specific Inhibition of Cyclophilins from Toxoplasma gondii
Source: ACS Infect Dis. 2023 Jan 18;9(2):365–77. doi: 10.1021/acsinfecdis.2c00566 (PMC9926490; doi:10.1021/acsinfecdis.2c00566)
Supplement: Supplementary file 1 — id2c00566_si_001.pdf [file id2c00566_si_001.pdf]

**Supporting Information**

# Structural basis for cyclosporin isoform-specific inhibition of cyclophilins from *Toxoplasma gondii*

Filippo Favretto[1 #], Eva Jiménez-Faraco[2 #], Carolina Conter[1], Paola Dominici[1], Juan A. Hermoso[2 *], Alessandra Astegno[1 *]

[1]Department of Biotechnology, University of Verona, Strada Le Grazie 15, 37134 Verona, Italy.

[2] Department of Crystallography and Structural Biology, Institute of Physical Chemistry Rocasolano (IQFR), CSIC, Serrano 119, 28006 Madrid, Spain

[#]These authors contributed equally to this work.

*Corresponding authors. Alessandra Astegno: email: alessandra.astegno@univr.it. Juan A. Hermoso: email: xjuan@iqfr.csic.es

## Table of Contents

**Table S1.** Secondary structure content (%) of TgCyp18.4 and TgCyp23.
**Table S2.** Data collection and refinement statistics for TgCyp23:CsA complex.
**Figure S1.** Multiple sequence alignment to analyze the sequence of cyclophilin homologs of human CypA in *T. gondii*.
**Figure S2.** Properties of recombinant TgCyps.
**Figure S3.** Representative steady-state initial velocity kinetics for human CypA
**Figure S4.** Linear dependence of product formation on the concentration of Cyp enzymes.
**Figure S5.** TgCyp-CsA interaction.
**Figure S6.** Structural comparison among TgCyp23 homologues.
**Figure S7.** Structural comparison with *Homo sapiens* CypA.
**Figure S8.** Three-dimensional structure of CsCyp (pdb code: 4JJM) in complex with CsA.

**Table S1.** Secondary structure content (%) of TgCyp18.4 and TgCyp23.

| | **TgCyp18.4** | **TgCyp23** |
|---|---|---|
| Alpha-helix | 10.5 | 17.5 |
| Beta-sheet | 37.7 | 28.6 |
| Random coil | 51.8 | 53.9 |

**Table S2.** Data collection and refinement statistics for TgCyp23:CsA complex.

| | |
|---|---|
| *Data Collection*[a] | |
| Space group | P $2_1$ |
| Cell dimensions | |
| *a, b, c (Å)* | 38.40, 119.42, 46.35 |
| *α, β, γ (deg)* | 90, 103.62, 90 |
| Wavelength (Å) | 0.979181 |
| Resolution (Å) | 45.05- 1.10 (1.12-1.10) |
| unique reflections | 163283 (7984) |
| $R_{merge}$ | 0.040 (1.026) |
| $R_{pim}$ | 0.025 (0.676) |
| $CC_{1/2}$ | 1.000 (0.715) |
| mean $I/\sigma I$ | 16.8 (1.6) |
| Completeness (%) | 99.8 (99.0) |
| Multiplicity | 6.6 (6.3) |
| *Refinement* | |
| Resolution (Å) | 45.05-1.10 |
| $R_{work}/R_{free}$ | 0.1326/0.1530 |
| no. of atoms | |
| macromolecules | 3356 |
| solvent | 470 |
| *Ramachandran Analysis* | |
| Ramachandran favored (%) | 98.23 |
| Ramachandran outliers (%) | 0 |
| average B, all atoms (Å$^2$) | 19.0 |
| *Root-mean-square deviation (RMSD)* | |
| bond lengths (Å) | 0.0103 |
| bond angles (deg) | 1.5711 |
| ***Protein Data Bank entry*** | 8B58 |

[a]Values between parentheses correspond to the highest resolution shells.

```
hCypA         1    ----------------------------MVNPTVFFDIAVDGEPLGRVSFELFADKVPKTAENFRALSTGEKGF-G-------YKGSCFHRIIPGFMCQGGDFTRHN   71
TgCyp205700   170  -------------Y-------SSR-LSTMPNPRVFFDISIDKKPAGRIEFELFADVVPKTAENFRALCTGEKGT-GRSGKPLYYKGCPFHRIIPQFMCQGGDFTRMN   254
TgCyp230520   57   -----------QSG-------VLS-ELKMANPRVFFDIAIGGRPAGRVEFELFKSIVPRTVENFRALCTGEKGV-GVSGKPLCYKNSTFHRIIPSFMCQGGDFTRFN   143
TgCyp221210   2    --------KLVLLFLALAV-SGAV-AENAGVRKAYMDIDIDGEHAGRIILELREDIAPKTVKNFIGLFDK-------------YKGSVFHRIIPDFMIQGGDFENHN   85
TgCyp285760   22   ---------AGISYAEAMEGGSRP-LLHPDNPVVFFDISIGSHEAGRIKIELFKNLAPKSAENFRQFCTGEFR---QNQVPIGYKGATFHRIIKNFMIQGGDFVKGD   115
TgCyp238000   38   ----------PEAGRSAVA-DSGR-RSFLPNPLVHLDISIGGRDAGRMVFQLFADTHPITAENFRCLCTGETGL-GYWMRPRWYKSTPFHRIVPGFMCQGGDIHRGD   131
TgCyp283850   314  --------KKVDLYTE----KQGV-RNVSKCPKVYMDIKVGDNAPKRVVFALYNDTVPKTAENFRALCTGEKGE-GKKGKPLCFKNSLFHRVIPGFMMQGGDFTNGD   406
TgCyp229940   1    ----------------------------MGKTTVYLDISIGNKAGGRLVFELFTDITPKAAENFRGLCTGEYGLSGRTGKPLHYLGSSFFRIVPGVLIQGGDVQNND   79
TgCyp270560   10   ---SRKAVSSPSLQASLPSAASEGAGVYRGDETVTL-----VTSMGDVEVELYWKHAPKTCRNFFELAKSGY-----------YDNTVFHRVAKDFCIQGGDPT-GT   96
TgCyp320640   579  RDVFNEKPSKEEQEALAPSASSAIAPSLRSGRTATI-----FTTMGDIFIRLFPQECPKTVENFCTHARNGY-----------YDQCIFHRVIKGFMIQTGDPN-GD   668
TgCyp289250   1    -------------------------------MSVTI-----KTNLGDLKAELYCQQAPKTCKNFLALCASDY-----------YNGTRFHRNIKGFAIQGGDPT-GT   59
TgCyp262520   74   -----------RLQRD--RSVSAV-HKQAIRDRVFMDFALGGRYIGRVLIGLYSDRVPLSVENFIQLCEGYR----VQDKVIGYRNTPVHLVKRGVNLTAGDVLSGT   162
TgCyp305940   293  TQEFREL-----SEKERMQPIYARCRKLKKKGYLRI-----VTTQGSLNIELHADMAPRACDSFLRLCAVKY-----------FDDTIFHRCIRNFMIQGGRAE-LR   377
TgCyp241830   1    -----------------MSEVYVHEPS--TRGKVVL-----HTSLGDLDVELWARECPLACRNFVQLCLEGY-----------YVNTIFHRVVKDFIVQGGDPT-GT   71
                                                      :           :   *     * :  .*                   :    ..     .    *

hCypA              GTG----------GKS--IYGEKFEDENF-ILKHTGPGILSMANAG-P-------------NTNGSQFFICTAKTEWLDGKHVVFGKVKEG--MNIVEAMERFG--S   147
TgCyp205700        GTG----------GES--IYGEKFADENF-SYKHSEPFLLSMANAG-P-------------NTNGSQFFITTVPCPWLDGKHVVFGKVVAG--QEVVKMMEAEG--R   330
TgCyp230520        GTG----------GES--IYGRTFADENF-KLKHTEPFLLSMANAG-P-------------NTNGSQFFITTVPCPWLDGKHTVFGKVTSG--QNVIKMMESQG--T   219
TgCyp221210        GTG----------GHS--IYGRRFDDENF-DLKH-ERGVISMANAG-P-------------NTNGSQFFITTVKTEWLDGRHVVFGKITTE-SWPTVQAIEALG--G   161
TgCyp285760        GTG----------RLS--IYGSSFPDEAF-VLPHFRSGLLSLANSG-P-------------DTNGCQFFITCAKCDWLNRKHVVFGQVLGKESMQVVRKIEHVTV-D   194
TgCyp238000        GRG----------GEC--IYGQFFRDERF-LYKHSKRGLLSMAKARRQ-------------HTNNSQFFITFDACPWLDGEHVVFGQLQSG--AEVLDQIEEQG--T   208
TgCyp283850        GTG----------GES--IYGPQFNDEKF-VDQHTGRGQLSMANCG-P-------------NTNSSQFFITFGPAPHLDGKHVVFGEVVEG--QDVLDEVEDVETDK   484
TgCyp229940        GTG----------GEC--VWGGTFRDENF-VRRHAQAGCLAMANNG-R-------------HTNGSQFYITLKKASALDNRHVVVGQLVEG--MELLRAMQLVPIDA   157
TgCyp270560        GRG----------GES--IFGGKFEDEIHPDLRHTGAGVVSMANCG-P-------------DTNGSQFFICLAPAPFLDGKHSIFGRVKKG--METVKKISTVQT-T   174
TgCyp320640        GTG----------GES--IWGGDFEDEFHRALKHDRPFTVSMANAG-P-------------NTNGSQFFITTVPCSWLDNKHTVFGRVVQG--MDVVTKIENVAT-N   746
TgCyp289250        GKG----------GES--IYGGLFEDEFVSSLKHDRRGILSMANQGKP-------------NTNGSQFFITYCRQPHLNGVYSVFGRLIDG--MDTLDKMEREPV-G   138
TgCyp262520        GVG----------KLS--IYGLTFPDENF-DMQFIQDGDVALMNSG-P-------------STNNSQFIITFNALTPLNRNYVVIGTVLKG--MRVIKQIEEEAG-S   239
TgCyp305940        QPSKKKEVQQSPRSISGFPGGAPFEDEFDNRLVHQGIGVLSMANDGKH--------------SNLSEFFITFKSCEHLNNKHTIFGRVVGG--LDVLRQWEKLET-D   467
TgCyp241830        GRG----------GADTTFDGKPFDVETHPRLKFRYRGLVGVANLGRSSKDAENDERGRSLGTNGNQFFITLARADVLNNAYTLFGKVTGH-TLYNLMKFNDLEV-G   166
                     .                 *  *  *      .     :.: :                  :*  :* *       *:  : :.* :        :   .

hCypA              RNGKTSKKITIADCGQLE--------   165
TgCyp205700        SNGQPKCAVEISSCGQLS--------   348
TgCyp230520        QSGSVKQTVVITDCGETK--------   237
TgCyp221210        SGGRPSKVAKITDIGLLE--------   179
TgCyp285760        GGNRPRIPVTVTQCGEL---------   211
TgCyp238000        PGGWRKRPVEIWNCGELLLNTLREIP   234
TgCyp283850        SNDRPKQDVQIVDCGEVKC-------   503
TgCyp229940        KTGTPKVSIVIAGCGELGVAVRRLDA   183
TgCyp270560        ATDRPIYDIKIIRAVTA----TSLD-   195
TgCyp320640        VEDKPKQDVKLLTIKVTS--------   764
TgCyp289250        RKNRPENDIIIEQVIIHANPIAEMDQ   164
TgCyp262520        KTGAPMQPVRIINCGLYRGLQDGPPF   265
TgCyp305940        KKDKPLKPPKVEEIIVFKNPFEDARK   493
TgCyp241830        KEDRPMTPPFIKSVDVLWNPFEDLVP   192
                     .       :
```

**Figure S1.** Multiple sequence alignment to analyze the sequence of cyclophilin homologs of human CypA in *T. gondii.* The sequences were aligned using Clustal Omega.

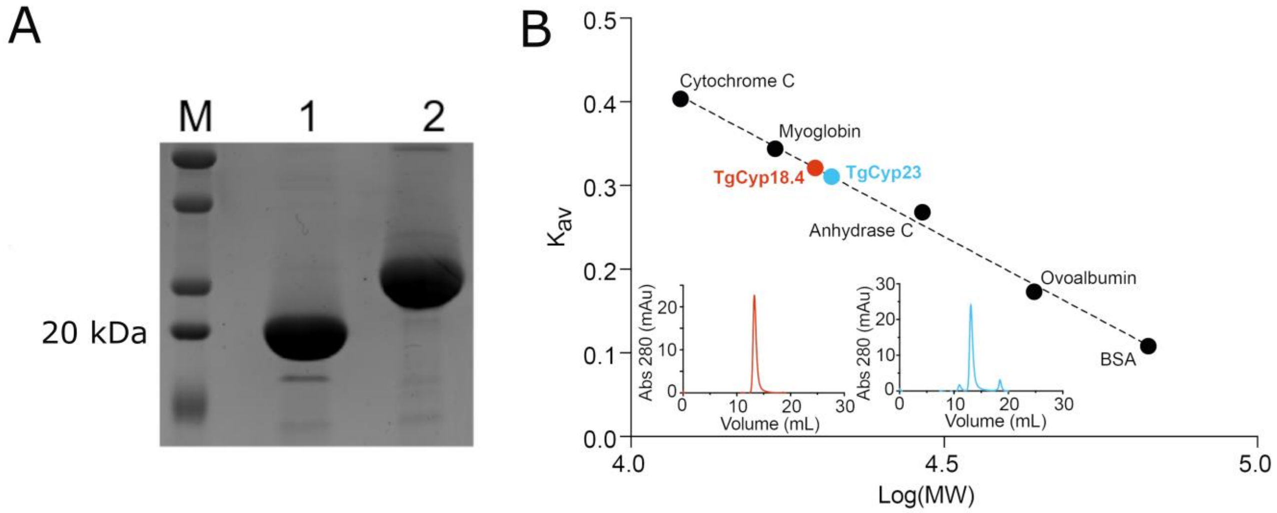


**Figure S2. Properties of recombinant TgCyps.** (**A**) 12% SDS–PAGE analysis of purified recombinant TgCyps. Lane M, protein marker, lane 1, TgCyp18.4, lane 2, TgCyp23. (**B**) Gel filtration chromatography of TgCyps at 1 mg/mL using Superdex 75 Increase 10/300 GL column (GE Healthcare) in 20 mM sodium phosphate, 150 mM NaCl buffer pH 7.5.

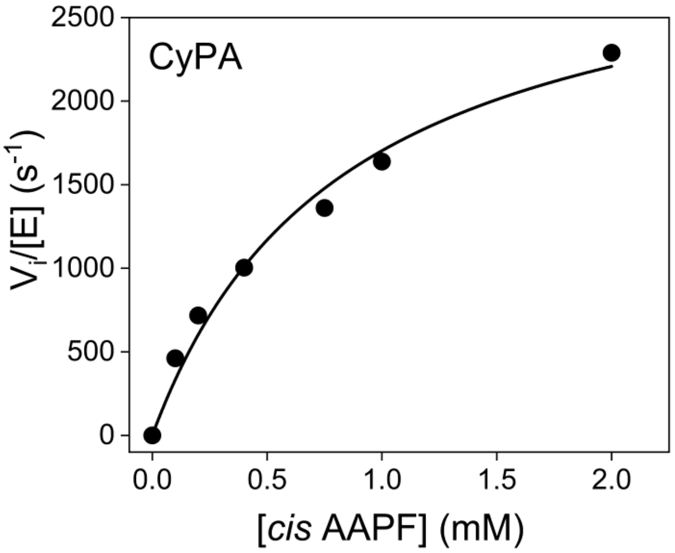


**Figure S3. Representative steady-state initial velocity kinetics for human CypA.**

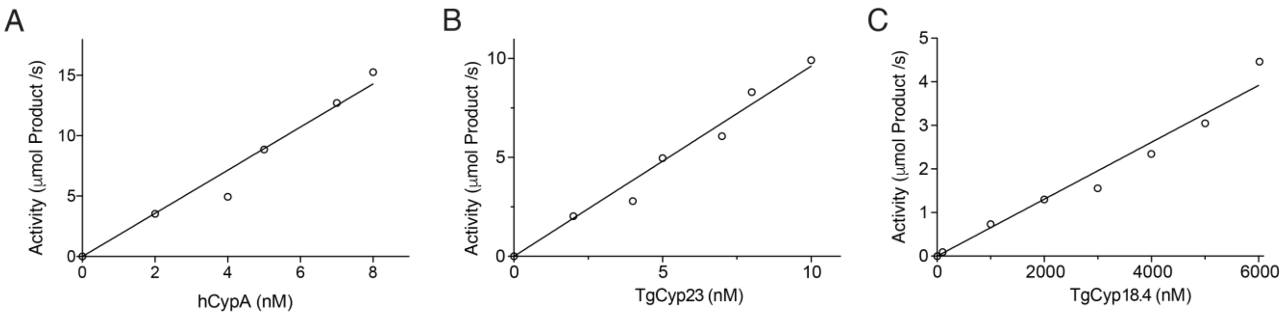


**Figure S4. Linear dependence of product formation on the concentration of Cyp enzymes.**

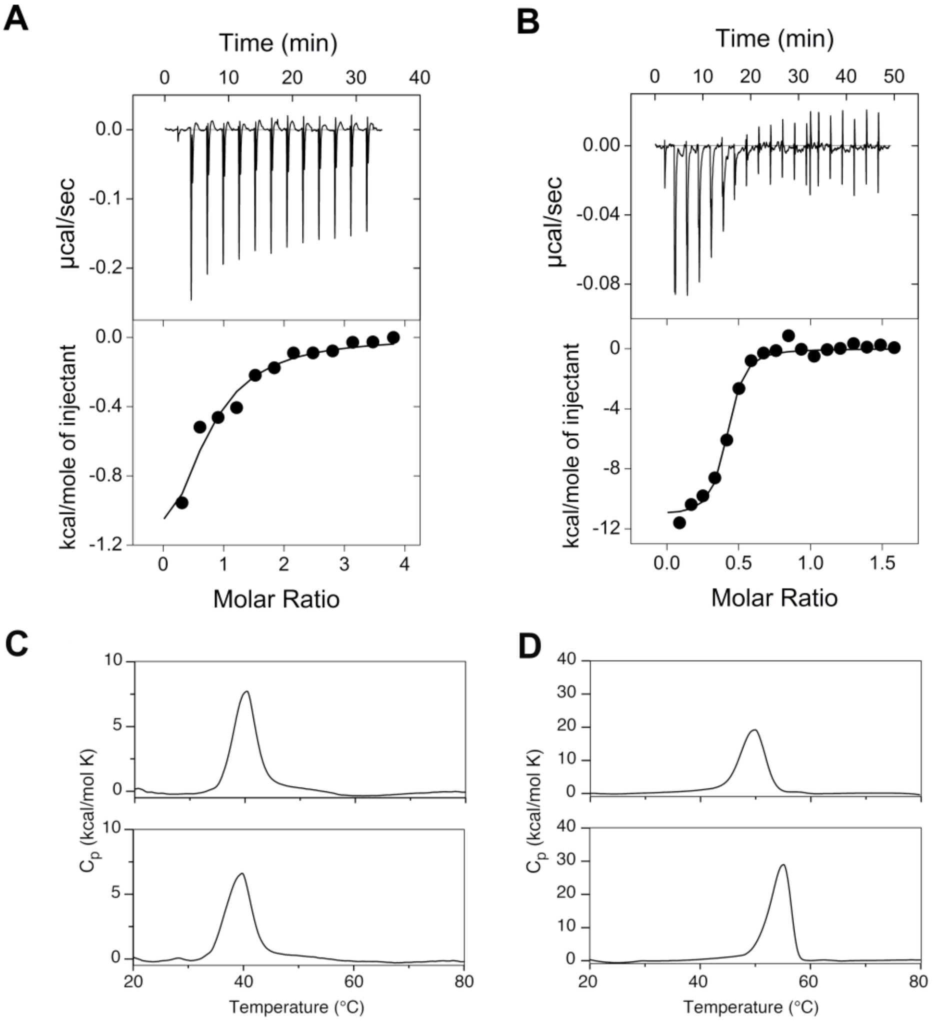


**Figure S5. TgCyp-CsA interaction (A-B)** Representative calorimetric titrations of CsA binding to TgCyp18.4 (**A**) and TgCyp23 (**B**) at 20 °C. The heats were determined by integration of the injection peaks (top panel) and the resulting titration curve (lower panel) was fitted to a single binding site model by non-linear least-squares analysis. **(C-D)** Thermal denaturation of TgCyp18.4 (**C**) and TgCyp23 (**D**) analyzed by DSC in the absence and presence of CsA. The scan rate was 1°C/min and the protein concentration was 70 μM.

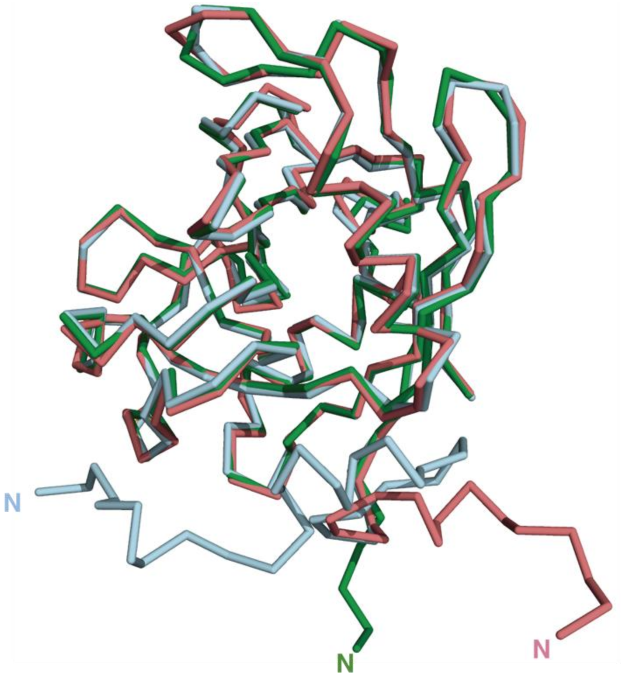


**Figure S6. Structural comparison among TgCyp23 homologs**. Structural alignment among TgCyp23 (blue), human U4/U6 snRNP-specific Cyp (PDB code 1MZW) (green), and *Plasmodium yoelii* Cyp (PDB code1Z81) (salmon). These structures are represented in ribbon. N indicates the N-terminus region.

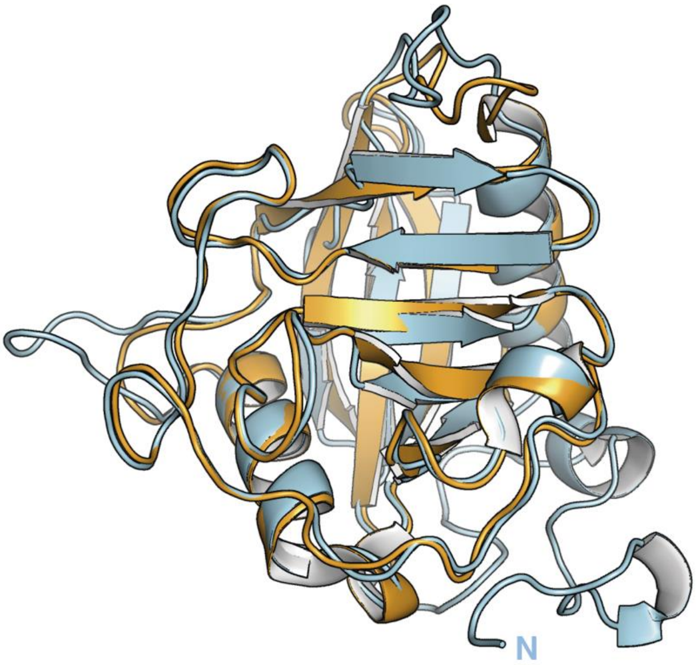


**Figure S7. Structural comparison with *Homo sapiens* CypA**. Alignment between CypA (yellow) and TgCyp23 (blue). Structures are displayed in cartoon view.

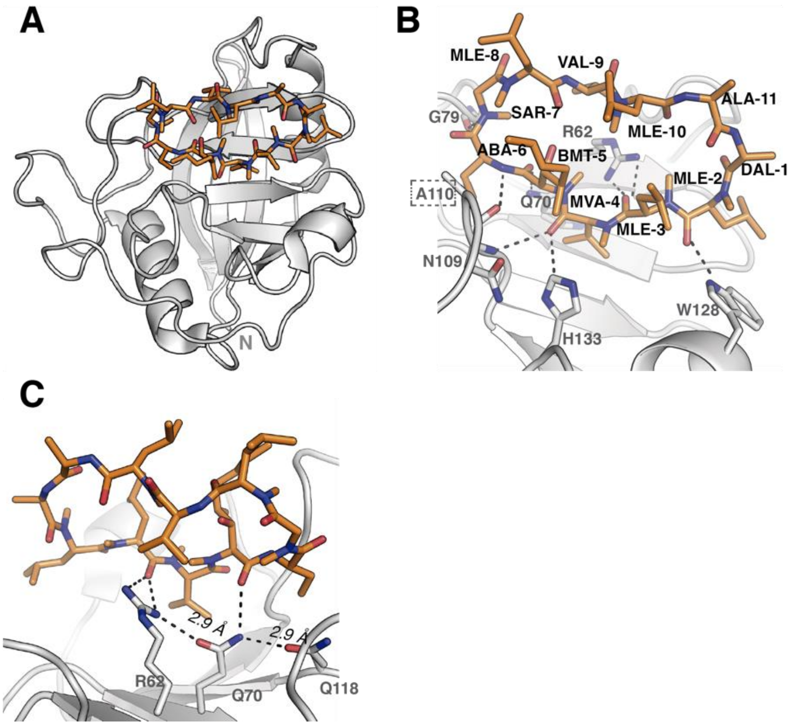


**Figure S8. Three-dimensional structure of CsCyp (PDB code 4JJM) in complex with CsA.** (**A**) (left) Overall structure; cyclophilin is displayed in gray cartoon and CsA is depicted as sticks. N indicates N-terminus region. (**B**) (right) Main interactions of the complex CsCyp-CsA. Relevant residues implicated in the interaction are labeled and shown as sticks. (**C**) Interaction network between nearby protein amino acids, (distance in angstroms are indicated). Dashed lines indicate polar interactions.
